# Efficient Deep Network Architectures for Fast Chest X-Ray Tuberculosis Screening and Visualization

**DOI:** 10.1038/s41598-019-42557-4

**Published:** 2019-04-18

**Authors:** F. Pasa, V. Golkov, F. Pfeiffer, D. Cremers, D. Pfeiffer

**Affiliations:** 1 Chair of Biomedical Physics, Department of Physics and Munich School of BioEngineering, Technical University of Munich, 85748 Garching, Germany; 2 Department of Diagnostic and Interventional Radiology, Klinikum rechts der Isar, Technical University of Munich, 81675 München, Germany; 3 Chair for Computer Vision & Artificial Intelligence, Department of Computer Science, Technical University of Munich, Boltzmannstrasse 3, 85748 Garching, Germany

**Keywords:** Health care economics, Radiography, Computational science

## Abstract

Automated diagnosis of tuberculosis (TB) from chest X-Rays (CXR) has been tackled with either hand-crafted algorithms or machine learning approaches such as support vector machines (SVMs) and convolutional neural networks (CNNs). Most deep neural network applied to the task of tuberculosis diagnosis have been adapted from natural image classification. These models have a large number of parameters as well as high hardware requirements, which makes them prone to overfitting and harder to deploy in mobile settings. We propose a simple convolutional neural network optimized for the problem which is faster and more efficient than previous models but preserves their accuracy. Moreover, the visualization capabilities of CNNs have not been fully investigated. We test saliency maps and grad-CAMs as tuberculosis visualization methods, and discuss them from a radiological perspective.

## Introduction

Tuberculosis is classified as the fifth leading cause of death worldwide, with about 10 million new cases and 1.5 million deaths per year^1^. Being one of the world’s biggest threats and being rather easy to cure, the World Health Organization recommends systematic and broad use of screening to extirpate the disease. Posteroanterior chest radiography, in spite its low specificity and difficult interpertation^1^, is one of the preferred tuberculosis screening methods. Unfortunately, since TB is primarily a disease of poor countries, the clinical officers trained to interpret these chest X-Rays are often lacking^2,3^.

In these settings, an automated algorithm for tuberculosis diagnosis could be an inexpensive and effective method to make widespread tuberculosis screening a reality. As a consequence, the topic has attracted the attention of the machine learning community, which, in a range of publications^3–12^, tackled the problem with methods ranging from hand-crafted algorithm to support vector machines and convolutional neural networks. The results are encouraging, as some of these methods achieve nearly-human sensitivities and specificities.

Over the last years, end-to-end trained convolutional neural networks (CNNs) have shown drastically superior performance on a multitude of image analysis challenges when compared to more classical hand-crafted algorithms or even other machine learning approaches such as support vector machines, in particular when the challenge can be sufficiently well characterized by abundant labeled training data. This makes deep learning a promising approach for medical image analysis^9^ and showed state-of-the art performances in tasks spanning from breast cancer classification^13^, organ^14^ and tumor segmentation^15,16^ to scan time reduction for diffusion MRI^17^, to name a few.

The use of deep learning on chest X-Rays has attracted some attention^18,19^ due to the cheapness of this imaging technique, the abundance of data^20^ and the similarity to natural images, which allows the transfer of models to medical tasks. The effectiveness of these algorithms on chest x-ray data has been shown in various publications, with some even generating automatic annotations for the symtoms^18^.

For the case of tuberculosis diagnosis, deep convolutional networks have demonstrated performances at least on par with those of the best competing approaches, while being conceptually simpler. Competing approaches often make use of complex machine learning pipelines^3,4,8,11^. For instance, Vajda *et al*.^11^ use a pipeline that starts with an atlas based lung segmentation algorithm, then extracts manually selected features such as shape and curvature descriptor histograms or the eigenvalues of the hessian matrix, and finally uses a classifier to diagnose the disease. They obtain results on par with results using deep learning^6^, but their multi-stage pipeline is more complex that an end-to-end trained convolutional neural network and requires more development work.

Previous publications using deep learning adopt deep learning models such as AlexNet, GoogLeNet and ResNet, which were developed for natural images classifications tasks^5–7,18^. While these are very powerful classifiers, they have been developed and optimized to be trained on more than a million images and to distinguish between a thousand classes. As a consequence, they require large amounts of memory and computation, both for training and inference, and their large number of degrees of freedom makes them prone to overfitting and less likely to generalize well when applied to medical tasks with limited amounts of data^21^.

Additionally, previous studies tackle only superficially the task of visualization^6^, which is important to assess the limitations of such techniques from a radiological perspective and to provide a second opinion to radiologists. A deeper understanding of the radiological aspects could be helpful for further advances and to build trust among the medical community in light of a future practical application.

In the present work we address these issues. We present a deep learning architecture tailored to tuberculosis diagnosis. With this approach we reduce the computational and memory requirement significantly, without sacrificing the classification performance. We further discuss the results of the training through the use of saliency maps and grad-CAMs. These techniques, which, to the best our knowledge, were never applied to this problem, provide an approximate visual diagnosis that might be a useful additional tool for clinicians.

## Methods

### Classification

**Figure 1:**
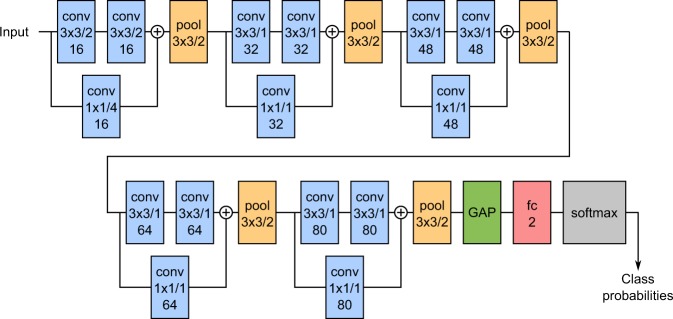
Schematic representation of the network architecture. conv = convolution, pool = pooling, GAP = Global Average Pooling, fc = fully connected. Convolutions and pooling sizes are reported as height × width/stride. The additional number indicates the number of feature maps for convolutions and the number of output neurons for the fully connected layer. Circled pluses indicate an addition operation.

The architecture of our network is shown schematically in Fig. 1. It consists of 5 convolutional blocks, followed by a global average pooling layer (which compresses each feature map to its mean value) and a fully-connected softmax layer with two outputs. Each convolutional block contains two 3 × 3 convolutions with ReLUs, followed by a max-pooling operation. The pooling size is 3 × 3 with stride 2, similarly to AlexNet^22^. Each block also has a shortcut connection implemented by a 1 × 1 convolution: the output of the shortcut is summed to that of the 3 × 3 convolutions just before pooling. The convolutions are all zero-padded in order to preserve the input resolution. Each convolutional layer also makes use of batch normalization^23^ to speed up the training procedure and reduce overfitting.

The convolutions of the first block have a stride of 2 in order to reduce the amount of computation required by the network. The resolution of the input is reduced by a factor of 8 by the first block, allowing us to use 512 × 512 images as input. A similar strategy is used by AlexNet^22^. The shortcut connection of the first block has stride 4 to match the resolution of the other convolutions. In our tests, using a stride of 2 had no effect on accuracy while decreasing the computational requirements significantly. Remarking that the first layer extracts very basic features, such as edges and stripes patterns^24^, a possible explanation for this is that the patterns can be extracted as easily with strided convolutions as with dense ones.We might as well invest the computational resources deeper in the network, where the represented features are much more complex and dense convolutions can be used more efficiently.

The depth of 5 blocks was chosen because this number corresponds to receptive window that covers the whole input image, and this window size allows the network to access a large context for its decisions at each location. The receptive window is the region around each location that can contribute to the activation of the neuron at that location. Empirical tests confirmed that this depth leads to good classification performances.

The input data is preprocessed with the following steps: (1) any black band or border is cropped from the edges of the image, (2) the image is resized so that the smaller edge is 512 pixels long and (3) the central 512 × 512 region is extracted. After this, the mean over all pixels in the whole dataset is subtracted and the pixel values are divided by their standard deviation.

Training was performed using categorical cross-entropy as the error function and with mini-batches of 4 samples. The samples are shuffled each epoch before forming the mini-batches, in order to randomize the whole learning procedure and reduce overfitting. The samples are augmented with elastic deformations with 80% probability. To deform the images, we lay a 4 × 4 square grid (e.g. with the tile side 128 pixels long) on the image and randomly displace the central grid vertices with random vectors whose component are sampled from a normal distribution with standard deviation of 32 pixels. The image is then interpolated with linear interpolation according to the displaced grid’s reference system^25^. We found deformations with small grid sizes but relatively high displacements to generate more realistic images than fine grids with small displacements, because fine grids tend to introduce a lot of small-scale deformation (i.e. ribs become wiggled).

For the weight initialization we used He normal initialization^26^ on all layers (in the last layer we divided the variance by $\sqrt{2}$ compared to the previously cited paper, because it uses softmax instead of rectification^27^). We trained for 150 epochs with the Adam optimizer^28^ using the following parameters: ${\beta }_{1}=0.9$, ${\beta }_{2}=0.999$, ${\epsilon }=1\times {10}^{-8}$ and learning rate $8\times {10}^{-5}$.

Our implementation of this architecture is available for download at https://github.com/frapa/tbcnn.

### Visualization

Once the network was trained for classification, we also generated saliency maps^24^ and gradient class activation maps (grad-CAM)^29,30^. These visualization techniques help us understand the network and may also be useful as an approximate visual diagnosis for presentation to radiologists.

Saliency maps and grad-CAMs generate a heatmap that shows which region of the image weights more for the classification. The principle these visualizations are based is the following: the derivative of the output class score w.r.t to an activation in a feature map indicates the impact this activation has on the class score. If the derivative is small, then a change in the activation will have a negligible impact on the output score, therefore the activation is unimportant for the classification. On the contrary, a big derivative indicates that the activation is important for the class score. Since the units in a feature map are spatially arranged, calculating the derivative for each unit generates an image.

The two techniques differ in how the derivative is back-propagated through the ReLUs, and in which feature map is used. Saliency maps calculate the derivative w.r.t. the input image, and thus generate a heatmap with the same resolution of the input. Grad-CAM use deeper feature maps, which typically results in better localization^29^ due to the higher-level nature of the features in deeper layers, but are available only at reduced resolution due to pooling. This is a trade-off which may or may not lead to better results.

We implemented these two visualizations as described in the original papers^29,30^. All the saliency maps and grad-CAMs are generated for the positive class, because we want to visualize the regions where tuberculosis in present and not the other way around.Materials

In our numerical experiments we used data coming from two different public databases: (1) the NIH Tuberculosis Chest X-ray dataset^31^, which is subdivided in two separate datasets from Montgomery County in Maryland and Shenzhen, and (2) the Belarus Tuberculosis Portal dataset^32^. The Montgomery and Shenzhen dataset contain 138 and 662 patients respectively, with and without TB, while the Belarus dataset has a total of 304 chest x-ray images of patients with confirmed TB. Table 1 shows some more information about the datasets.

**Table 1:** Overview of the datasets used for training and evaluation of tuberculosis classification.

	Montgomery	Shenzhen	Belarus	Total
Patients	138	662	304	1104
without TBC	80	326	0	406
With TBC	58	336	304	698
Male	63	442	194	699
Female	74	213	110	397
Other/Unknown	1	7	0	8
Cross-validation subset	27–28	132–133	—	220–221

The last row indicates the size of a subset used for the cross-validation study, which is about one-fifth of the total size of the dataset. It also represents the size of the validation and test sets.

We trained our network on the dataset from Maryland and Shenzhen for comparison with other publications. We also trained on the combination of all the three datasets to exploit all the available data and to take advantage of the differences in acquisition between the different datasets, which allows the network to learn more robust features.

## Results and Discussion

### Classification performance

A 5-fold cross-validation study was performed on each of our three datasets (Maryland, Shenzhen and Combined). The cross-validation study is very useful to estimate the accuracy of the model especially if the dataset is small (e.g. in the case of the Montgomery dataset), because the performances can change significantly between test sets. If many outliers are chosen to be in the test set, then the performances might be unsatisfactory because the neural network cannot learn to predict them from the training set. On the contrary, if most outliers are in the training set, then they do not count in our accuracy measurement and we might overestimate the quality of the model. Averaging among sets ensures the results are reliable.

**Figure 2:**
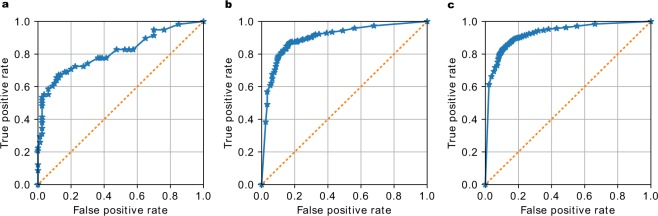
Receiver Operation Characteristic (ROC) curves of the 5-fold cross-validation study on three different datasets. The accuracy and Area Under the ROC (AUC) score are (**a**) 0.811 for the Montgomery dataset, (**b**) 0.9 for the Shenzhen dataset and (**c**) 0.925 for the combined dataset. For a description of the three datasets refer to Table 1.

The Receiver Operation Characteristic (ROC) curves for the three experiments are shown in Fig. 2. The accuracy and Area Under the ROC curves (AUC), shown in Table 2, are in line with the results in many other publications, which demonstrates that our architecture choice does not impact the classification performance. Note that in Table 2 we try to make the fairest comparison possible, including the best results on the exact same datasets from the cited papers, but excluding methods like pre-training on ImageNet and ensemble methods.

**Table 2:** Accuracy and AUC on the Maryland (MC), Shenzhen (SZ) and combined (CB) datasets withcomparison to other publications (cited in the second row).

	MC	SZ	CB	MC			SZ				Other^†^
				5	4	11	5	4	7	11	6
Accuracy [%]	79.0	84.4	86.2	67.4	78.3	78.3	83.7	84.1	88.0	95.6	—
AUC	0.811	0.900	0.925	0.884	0.869	0.87	0.926	0.900	0.91	0.99	0.94–0.95

Among the various results reported in these publications, we selected the best ones which are comparable to our results, for example excluding those that make use of models pre-trained on the ImageNet database. ^†^This dataset is similar to the combined dataset used here, as it uses part of the same data and has a similar number of patients.

If we do not restrict ourselves to the Montgomery and Shenzhen datasets and we include also ensemble methods and pre-training, we can find better accuracies and AUC scores than ours. Among other publication not using convolutional networks, Chauhan *et al*.^10^, for example, obtain 94.2% accuracy and AUC of 0.957: interestingly they use an SVM classifier based on whole-image features. Another example is the work is of Vajda *et al*.^11^, whose results are significantly better than ours, at 95.6% accuracy and 0.99 AUC for the Shenzhen dataset. Their approach consists in segmenting the lungs with an atlas-based algorithm, extracting features such as shape descriptor histograms and using a simple neural network for classification. The disadvantages of this method, apart from being rather complicated, is the need for annotated lung shapes and the use SIFT for segmentation, which is a patented algorithm for edge-detection and therefore not freely usable.

Among papers making use of CNNs, Hwang *et al*.^5^ obtain 90.3% accuracy and AUC of 0.964 using they use transfer learning from ImageNet and training on a dataset of 10848 CXRs. However, the accuracy and AUC they obtain without pre-training are lower than ours, despite having a database ten times larger. Training the network presented here on a database of this size is likely to outperform these results.

Lakhani and Sundaram^6^ of the other hand, use the well-known AlexNet and GoogLeNet with excessive augmentation and without pre-training to reach an AUC of 0.94-0.95. Using transfer learning and an ensemble of the two networks they achieve an AUC of 0.99. A similar approach is used by Islam *et al*.^7^: in their work the AlexNet, VGG and ResNet models are compared. Only ResNet, the most recent and powerful model, achieves slightly better accuracies (88% versus 86.2%) but with lower AUC (0.91 vs 0.925) than the present work. They also build an ensemble of 6 CNNs and obtain 90% accuracy and 0.94 AUC.

It seems plausible that, increasing the amount of training data or performing pre-training on a bigger dataset, it would be possible to obtain similar results with our architecture. The use of pre-training is however out of the scope of this work.

### Time and size requirements

The advantage of this network is that it has only about 230,000 parameters, whereas the second smallest network (GoogLeNet) has 7 million^33^. Other architectures used in the studies cited above have up to 60 million parameters^22^. Therefore, the architecture presented here has the highest parameter efficiency. A more compact network with fewer degrees of freedom is less prone to overfitting and more likely to generalize well, which is confirmed by the fact that the only regularization measures used were batch normalization and data augmentation.

The number of parameters and the complexity of the network affect the training and inference speed and its hardware, energy and memory requirements. Our best network can be trained in about 1 hour on a low-end Nvidia GeForce GTX 1050 Ti, which currently (as of 2019) costs less than $ 200. Training requires about 800 MB of graphic memory. The inference on the same GTX 1050 Ti takes about 5–6 milliseconds and uses less than 200 MB of memory. Inference requires less computational power and memory than training, therefore the neural network could even be deployed on much cheaper, less powerful and energy hungry hardware, such as a low-cost board computer like a Raspberry Pi, while keeping the inference time acceptable for the use case.

A case for simpler and more efficient methods has already been made by previous publications^10–12^, since it would make it easier to deploy these models cheaply and effectively, for low resource regions where tuberculosis is more dominant. Unfortunately, no other publication reports processing time and memory requirements. We will nonetheless attempt a comparison with other works, using the number of floating-point operations, or FLOP, as a meter for speed. Such comparison is not always proportional to actual performances, but is useful as a rough estimate.

Our architecture has about 350 MFLOPs and 230 thousand parameters. The AlexNet used by Lakhani and Sundaram^6^ has 1.5 GFLOPs and about 60 million parameters, while GoogLeNet, used by the same authors, has about 3 GFLOPs and 7 million parameters. ResNet 152, which is the model we report in Table 2 for Islam *et al*.^7^, has about 11 GFLOPs and 60 million parameters. The only work not using a standard architectures is those of Hwang *et al*.^5^: the model reported in their work has about 11 GFLOPs. As a confirmation for this fact, they report using a top-of-the-line Nvidia Titan with 12 Gb of memory, while training on 500 × 500 images instead of 512 × 512 and having similar accuracy. Moreover, the ensemble methods used by some of these papers increase the requirements still further.

The publications making use of other machine learning techniques are harder to compare without reproducing the experiments, partly due to missing information in the publications but also because of differences in the datasets and hardware used. Karargyris *et al*.^12^ report that splitting their classifier leads to faster processing. The similar paper of Vadja *et al*.^11^ use a limited set of features to improve accuracy and reduce the inference time. How they both compare to our results is unclear: we can only guess that inference for the work of Vadja *et al*.^11^ could take more than 1 second, because the SIFT algorithm they use for edge detection has been reported to have such performances in similar cases^34^. A more promising approach is the one of Chauhan *et al*.^10^, because they skip the segmentation entirely, but they unfortunately do not report any performance information. Melendez *et al*.^3^ report their training time using an SVM, their best method has a training time of about 3 hours, about 3 times ours, while having slightly lower accuracy and training on only 300 images.

Therefore, our architecture achieves at least a 4-fold reduction in computational complexity compared to other deep learning models, and does it without affecting classification accuracy. A general comparison with other techniques is difficult, but seems to indicate that our approach is competitive. We believe the main advantage of our work is that a smaller network allows the use of cheaper and less power-hungry hardware, which could help the adoption of these algorithms, especially in low-income countries and remote locations.

### Visualization

In this section we discuss some selected saliency maps and grad-CAMs, and argue that they are useful tools to visualize tuberculosis. The samples presented in the following paragraphs have been generated with the network trained on the Montgomery dataset, unless noted. In the supplementary materials we provide about ten random saliency maps generated for each case (true positives and negatives and false positives and negatives) in order to prove the usefulness of this approach. All these supplementary saliency maps have been generated with the network trained only on the Montgomery dataset.

**Figure 3:**
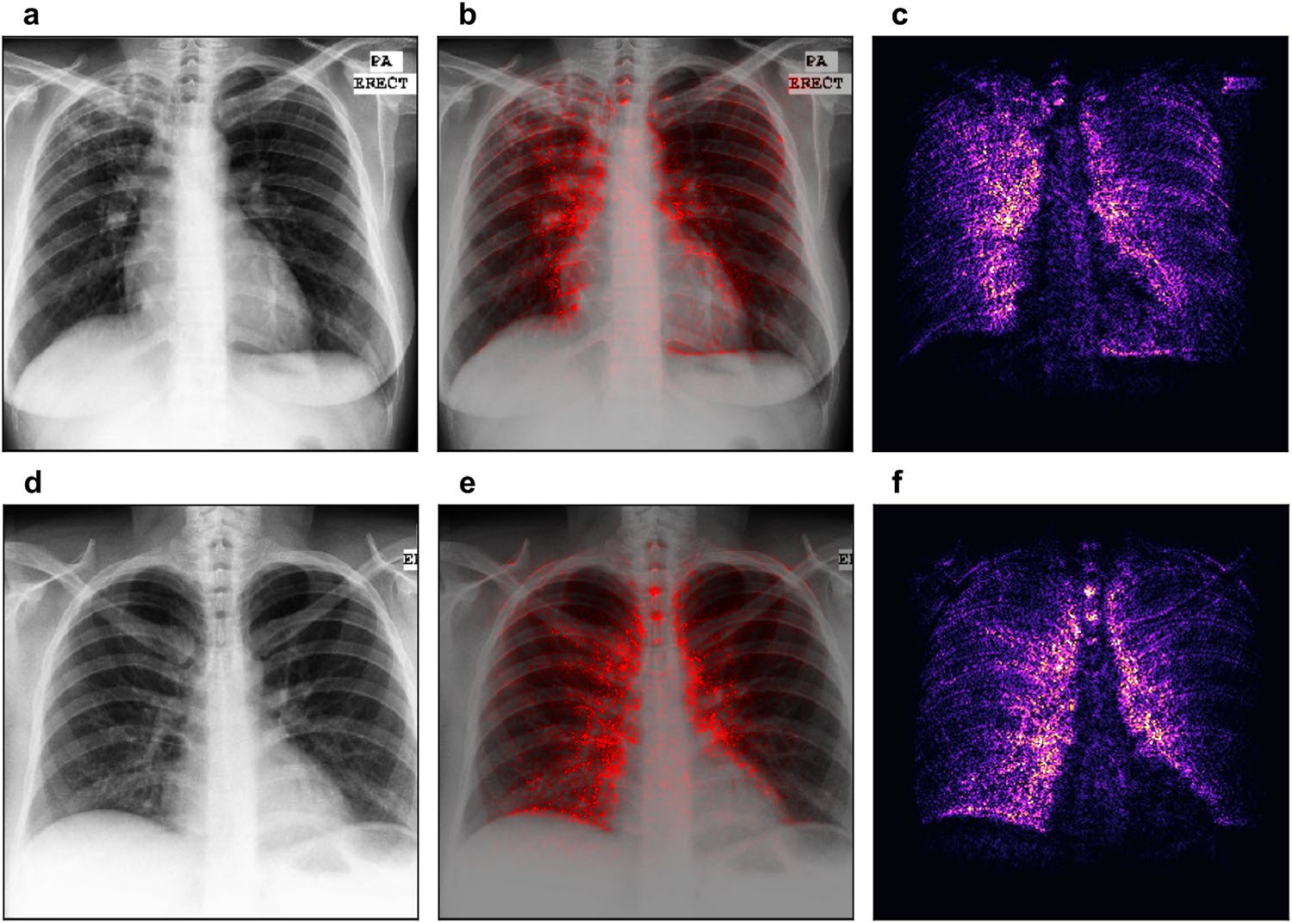
Saliency map with overlay for two correctly classified cases. Panels (a) and (d) show the chest images of the patients, panels (c) and (f) show the saliency maps, while panels (b) and (e) show the saliency maps overlaid on the chest images for comparison. The first row shows a patient with tuberculosis, with output score 0.98 (the maximum was 1). The second row shows a healthy patient with score 0.00 (the minimum was 0). Both scores suggest high confidence in the prediction.

The first row of Fig. 3 shows an example of a chest X-ray truly classified as positive for tuberculosis. The Chest X-ray shows patchy opacities in the right upper lobe with pleural apical thickening and upward deviation of the right hilum. These findings are consistent with pulmonary tuberculosis. In the saliency map, the outline of the soft tissue structures of the mediastinum are highlighted, and especially the area of the right upper lobe. This correlates perfectly with the pathological changes seen in the X-ray image.

The second row of Fig. 3 gives an example of the chest X-ray of a healthy patient, which was correctly identified as negative for tuberculosis. Saliency map (panel f) shows symmetric high lightening of the borders of the mediastinum. There is no increased signal in any of the lobes of the lung.

**Figure 4:**
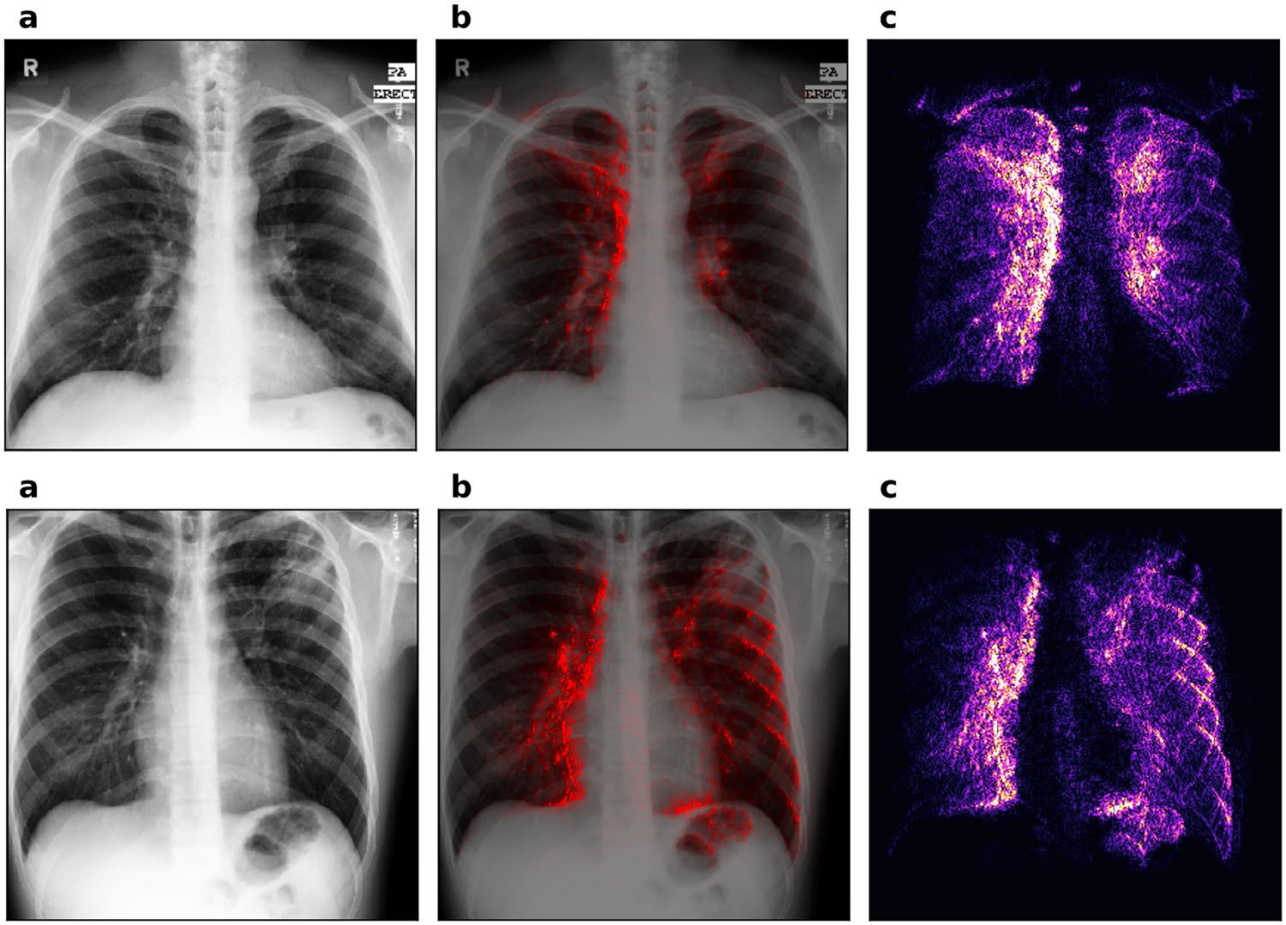
Saliency map with overlay for two misclassified patients. Panels (a) and (d) show the chest images of the patients, panels (c) and (f) show the saliency maps, while panels (b) and (e) show the saliency maps overlaid on the chest images for comparison. The first row shows a healthy patient classified with output score 0.98 (the maximum was 1). The second row shows a patient with tuberculosis with output score 0.04 (the minimum was 0). Both scores suggest serious misclassifications.

The first row of Fig. 4 shows a healthy patient, which was wrongly classified as positive for tuberculosis. The saliency map indicates an area in the right upper lobe as suspicious for tuberculosis. Indeed, this area presents with decreased radiolucency, however, this is caused by superposition of several bones, namely the clavicle and the ribs.

The patient in the second row of Fig. 4 suffers from tuberculosis; however, the algorithm rated this X-ray image as negative. There are patchy opacities and bronchiectasis in the left upper lobe of the lung. These findings are consistent with pulmonary tuberculosis. Saliency map shows a main focus on the border of the mediastinum on the right and also the edges of several ribs on the left hemithorax.

In general, neural networks usually identify edges and use this information for detection of findings and decision-making. In our examples, the saliency maps always highlight the edges between the organs of the mediastinum, which present with a high attenuation, and the transparent lung tissue.

**Figure 5:**
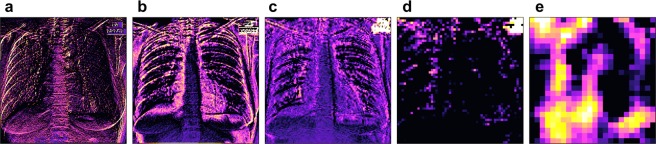
Gradient class activation maps (grad-CAMs) for different layers of the same patient. Panel (a) shows layer 1, (**b**) layer 4, (**c**) layer 7, (**d**) layer 10 and (**e**) layer 13. The scales of each panel are independent of each other. The patient is also shown in the first row of Fig. 3 (the true positive). The activation maps are calculated for the positive class and are shown for the last layer of each convolutional block (e.g. the one just before pooling). Activation maps of the higher layers show higher level features, which should be tuberculosis specific.

Figure 5 shows the grad-CAMs of different layers for one on the correctly classified patients. All the images were generated for the positive class in order to highlight features that are important for the detection of TB. Grad-CAMs can be generated for each layer in the network and have been reported to offer better localization than saliency maps for natural image recognition tasks^29^. However, one characteristic of grad-CAMs is that they show better localization ability when generated on deeper layers that represent higher-level features, but for these layers they are also of lower resolution due to pooling. Since tuberculosis’ features are typically rather small, we found that grad-CAMs are not as useful as saliency maps for diagnostic purposes.

**Figure 6:**
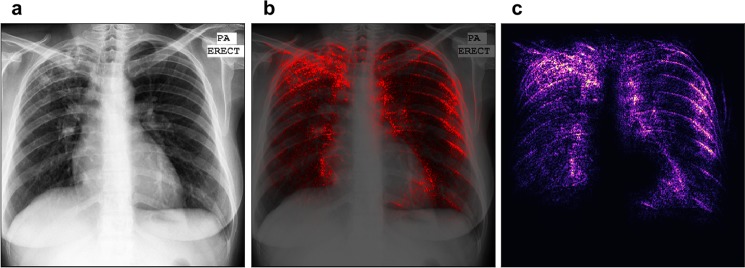
Saliency map of our network trained on the combined dataset, using about 660 patients for training. Panels (a) shows the chest images of the patients, panel (c) shows the saliency maps, while panel (b) shows the saliency maps overlaid on the chest images for comparison. This figure should be compared to the first row of Fig., which shows the saliency map predicted for the same patient, but with the network trained only on the Montgomery dataset. The localization ability of this saliency map is drastically improved. The image appears less noisy in unimportant regions and more intense in the areas where tuberculosis is really present.

Qualitatively, the saliency maps benefit from the bigger dataset more than the sheer classification accuracy or AUC score. Figure 6 shows the saliency map for the patient which is also shown in the first row of Fig. 3, but produced using the network trained on the combined dataset. In this figure we can see that, in spite of a relatively low accuracy increase of 7%, the image appears much easier to interpret (compare with the radiologist’s diagnosis above). The focus of the saliency map is indeed on the tuberculosis-affected areas, while the noise and the disturbing edge-detection effect are reduced. This result is remarkable for its precision, if the absence of any annotation is taken into account. Localization seems to benefit from a bigger dataset more than other metrics.

## Conclusion

This work presents an advanced neural network architecture optimized for tuberculosis diagnosis. We can train this specialized architecture from scratch and achieve good results compared to other publications, while reducing the computational, memory and power requirements significantly.

We also analyzed the output with saliency maps and grad-CAMs and found that saliency maps offer a good visual explanation of the network decision. Saliency maps were interpreted by an expert radiologist (one of the authors, D.P.) and were found to highlight areas where tuberculosis was visible in many cases. We believe that saliency maps can be an important tool for clinical officers to review and interpret the decision making of the algorithm.

Open points that need to be addressed in future work include the use of pre-training and bigger datasets to bring the classification accuracy and AUC on par with other works, while preserving our speed advantage. Since the network is able to localize the symptoms of tuberculosis, it could even be possible to generate a textual annotation for each case, as was successfully done in similar publications^18^.

## Supplementary information

Samples of saliency maps

## Data Availability

The Montgomery and Shenzhen dataset used in this study are published by the U.S. National Institute of Health (NIH) and are accessible at https://ceb.nlm.nih.gov/repositories/tuberculosis-chest-x-ray-image-data-sets/31. The Belarus dataset is published by different Institutes in Belarus on the Belarus Tuberculosis Portal^32^, accessible at http://tuberculosis.by.

## References

[1] Anderson L (2015). WHO Global tuberculosis report 2015. WHO Libr. Cat. Data.

[2] Van’t Hoog AH (2011). High sensitivity of chest radiograph reading by clinical officers. Int. J. Tuberc. Lung Dis..

[3] Melendez J (2016). An automated tuberculosis screening strategy combining X-ray-based computer-aided detection and clinical information. Sci. Rep..

[4] Jaeger S (2014). Automatic tuberculosis screening using chest radiographs. IEEE Trans. Med. Imaging.

[5] Hwang S, Kim H, Jeong J, Kim H (2016). A Novel Approach for Tuberculosis Screening Based on Deep Convolutional Neural Networks. Proc. SPIE.

[6] Lakhani P, Sundaram B (2017). Deep Learning at Chest Radiography: Automated Classification of Pulmonary Tuberculosis by Using Convolutional Neural Networks. Radiology.

[7] Islam, M. T., Aowal, M. A., Minhaz, A. T. & Ashraf, K. Abnormality Detection and Localization in Chest X-Rays using Deep Convolutional Neural Networks. *CoRR* abs/1705.0 (2017).

[8] Lopes UK, Valiati JF (2017). Pre-trained convolutional neural networks as feature extractors for tuberculosis detection. Comput. Biol. Med..

[9] Litjens Geert, Kooi Thijs, Bejnordi Babak Ehteshami, Setio Arnaud Arindra Adiyoso, Ciompi Francesco, Ghafoorian Mohsen, van der Laak Jeroen A.W.M., van Ginneken Bram, Sánchez Clara I. (2017). A survey on deep learning in medical image analysis. Medical Image Analysis.

[10] Chauhan A, Chauhan D, Rout C (2014). Role of gist and PHOG features in computer-aided diagnosis of tuberculosis without segmentation. Plos One.

[11] Vajda, S. *et al*. Feature Selection for Automatic Tuberculosis Screening in Frontal Chest Radiographs. *J. Med. Syst*. **42** (2018). 10.1007/s10916-018-0991-9 29959539

[12] Karargyris A (2016). Combination of texture and shape features to detect pulmonary abnormalities in digital chest X-rays. Int. J. Comput. Assist. Radiol. Surg..

[13] Rouhi R, Jafari M, Kasaei S, Keshavarzian P (2015). Benign and malignant breast tumors classification based on region growing and CNN segmentation. Expert Syst. Appl..

[14] Roth, H. R., Lu, L., Farag, A., Shin, H. & Liu, J. DeepOrgan: Multi-level Deep Convolutional Networks for Automated Pancreas Segmentation. 1–12 (2015).

[15] Milletari, F., Navab, N. & Ahmadi, S.-A. V-net: Fully convolutional neural networks for volumetric medical image segmentation. In *3D Vision (3DV), 2016 Fourth International Conference on* 565–571 (2016).

[16] Ronneberger, O., Fischer, P. & Brox, T. U-net: Convolutional networks for biomedical image segmentation. In *International Conference on Medical Image Computing and Computer-Assisted Intervention* 234–241 (2015).

[17] Golkov, V. *et al*. q-Space Deep Learning: Twelve-Fold Shorter and Model-Free Diffusion MRI Scans. *IEEE Trans. Med. Imaging* **1057** (2015). 10.1109/TMI.2016.2551324 27071165

[18] Shin, H. *et al*. Learning to Read Chest X-Rays: Recurrent Neural Cascade Model for Automated Image Annotation. *IEEE Conference on Computer Vision and Pattern Recognition (CVPR),* pp. 2497–2506 (2016).

[19] Li, Z. *et al*. Thoracic Disease Identification and Localization with Limited Supervision. IEEE Conf. Comput. Vis. *Pattern Recognit*. 8290–8299 (2018).

[20] Wang, X. *et al*. ChestX-ray8: Hospital-scale Chest X-ray Database and Benchmarks on Weakly-Supervised Classification and Localization of Common Thorax Diseases. 2097–2106, 10.1109/CVPR.2017.369 (2017).

[21] Lu L (2016). Deep Convolutional Neural Networks for Computer-Aided Detection: CNN Architectures, Dataset Characteristics and Transfer Learning. IEEE Trans Med Imaging.

[22] Günther Johannes, Pilarski Patrick M., Helfrich Gerhard, Shen Hao, Diepold Klaus (2014). First Steps Towards an Intelligent Laser Welding Architecture Using Deep Neural Networks and Reinforcement Learning. Procedia Technology.

[23] Ioffe S, Szegedy C (2014). Batch normalization: Accelerating deep network training by reducing internal covariate shift. ICML.

[24] Simonyan, K., Vedaldi, A. & Zisserman, A. Deep Inside Convolutional Networks: Visualising Image Classification Models and Saliency Maps. Iclr 1- (2014).

[25] Simard PY, Steinkraus DD, Platt JC, Way OM, others (2003). Best practices for convolutional neural networks applied to visual document analysis. In ICDAR.

[26] He, K., Zhang, X., Ren, S. & Sun, J. Delving deep into rectifiers: Surpassing human-level performance on imagenet classification. Proc. IEEE Int. Conf. Comput. Vis. 2015 Inter, 1026–1034 (2015).

[27] Glorot X, Bengio Y (2010). Understanding the difficulty of training deep feedforward neural networks. Proc. 13th Int. Conf. Artif. Intell. Stat..

[28] Kingma, D. P. & Ba, J. Adam: A Method for Stochastic Optimization. 1–15. 10.1145/1830483.1830503 (2014)

[29] Selvaraju, R. R. *et al*. Grad-CAM: Why did you say that? Visual Explanations from Deep Networks via Gradient-based Localization. 1610.02391V2 1–5 (2016).

[30] Zhou, B., Khosla, A., Lapedriza, A., Oliva, A. & Torralba, A. Learning deep features for discriminative localization. in Proceedings of the IEEE Conference on Computer Vision and Pattern Recognition 2921–2929 (2016).

[31] Jaeger S, Candemir S, Antani S, Lu P, Thoma G (2014). Two public chest X-ray datasets for computer-aided screening of pulmonary diseases. Quant Imaging Med Surg.

[32] Belarus tuberculosis portal. Available at: http://tuberculosis.by.

[33] Szegedy, C. *et al*. Going Deeper with Convolutions. CoRR abs/1409.4 (2014).

[34] Panchal, P. M., Panchal, S. R. & Shah, S. K. A Comparison of SIFT and SURF. **1**, 323–327 (2013).

