# Supplementary material for: Efficient Deep Network Architectures for Fast Chest X-Ray Tuberculosis Screening and Visualization
Source: Sci Rep. 2019 Apr 18;9:6268. doi: 10.1038/s41598-019-42557-4 (PMC6472370; doi:10.1038/s41598-019-42557-4)
Supplement: Supplementary file 1 — Samples of saliency maps [file 41598_2019_42557_MOESM1_ESM.pdf]

Efficient Deep Network Architectures for Fast Chest X-Ray Tuberculosis Screening and Visualization

F. Pasa*[1,3], V. Golkov[3], F. Pfeiffer[1,2], D. Cremers[3], D. Pfeiffer[2]

[1]Chair of Biomedical Physics, Department of Physics and Munich School of BioEngineering, Technical University of Munich, 85748 Garching, Germany

[2]Department of Diagnostic and Interventional Radiology, Klinikum rechts der Isar, Technical University of Munich, 81675 München, Germany

[3]Chair for Computer Vision & Artificial Intelligence, Department of Computer Science, Technical University of Munich, Boltzmannstrasse 3, 85748 Garching, Germany

*Corresponding author. Correspondence to francescopasa@gmail.com

True positives

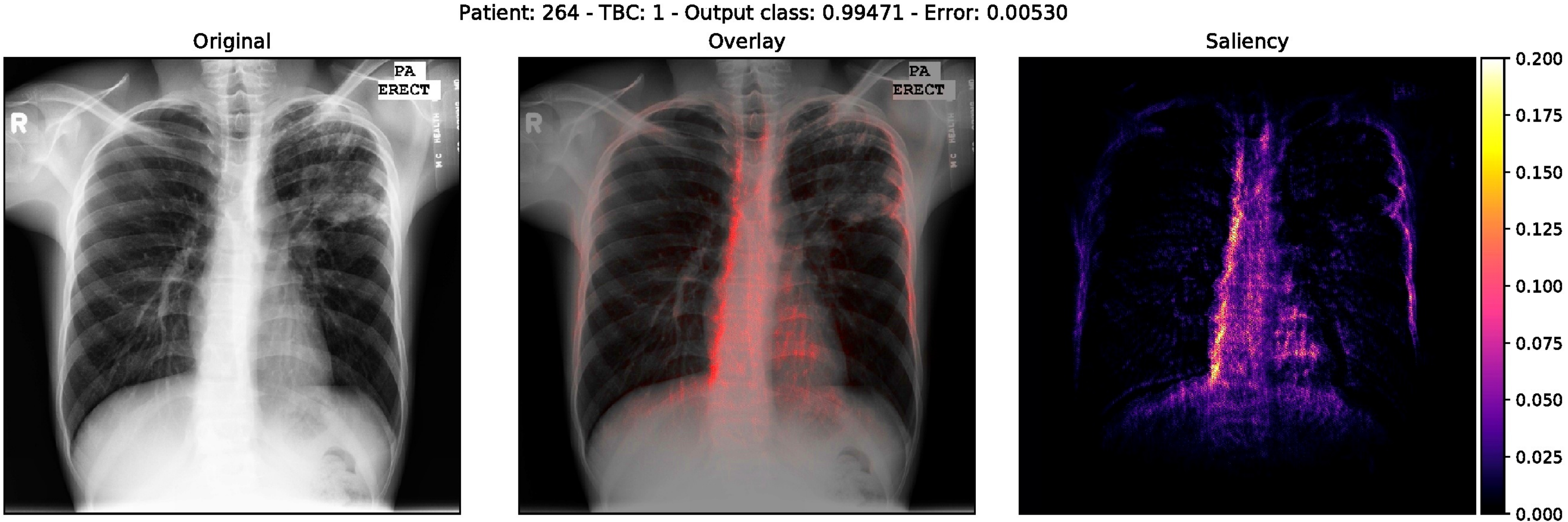
Patient: 264 - TBC: 1 - Output class: 0.99471 - Error: 0.00530
Original
Overlay
Saliency
PA
ERECT
R
0.200
0.175
0.150
0.125
0.100
0.075
0.050
0.025
0.000

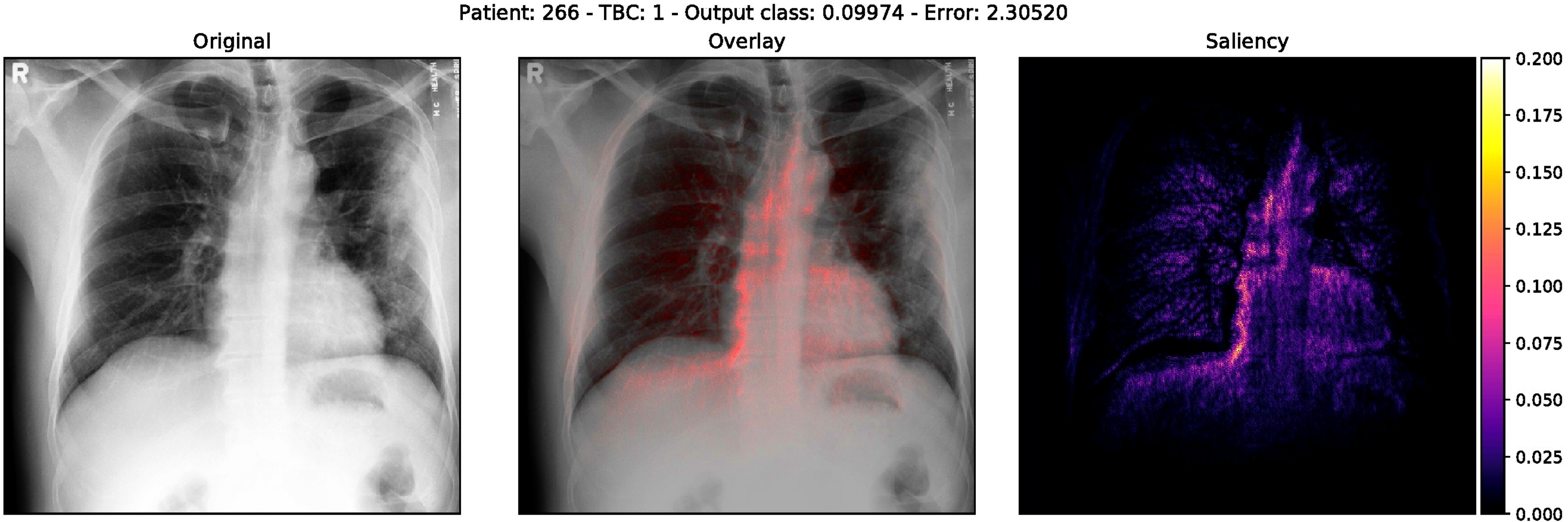
Patient: 266 - TBC: 1 - Output class: 0.09974 - Error: 2.30520
Original
Overlay
Saliency
0.200
0.175
0.150
0.125
0.100
0.075
0.050
0.025
0.000

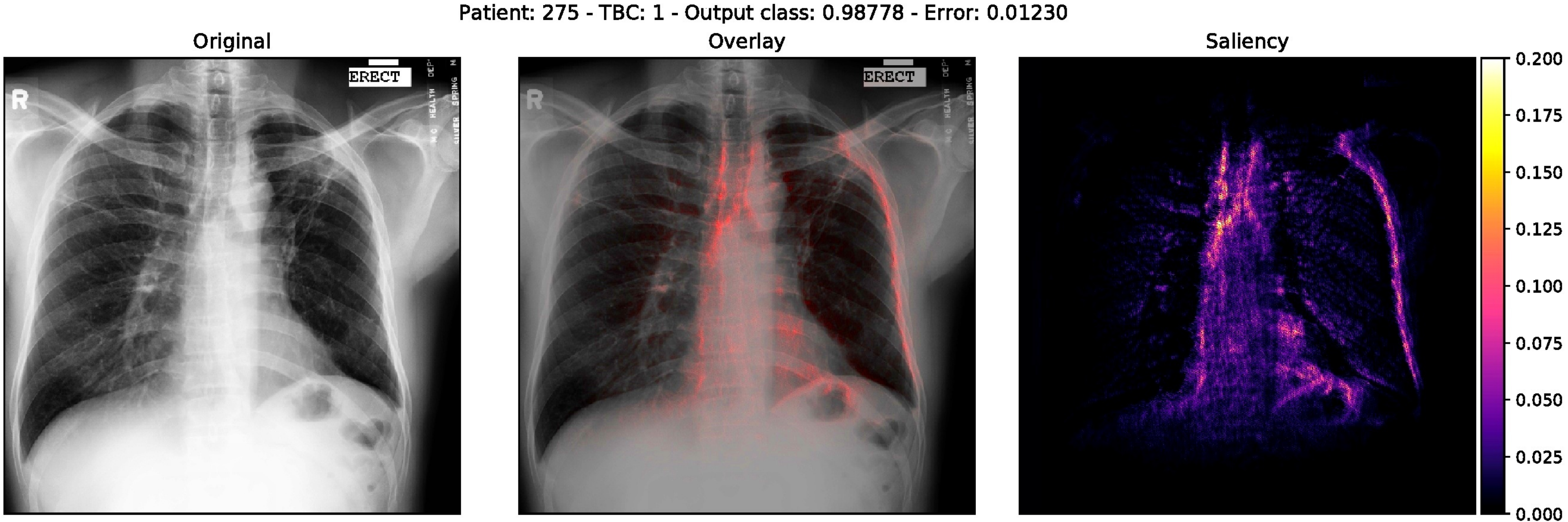

Patient: 275 - TBC: 1 - Output class: 0.98778 - Error: 0.01230
Original
Overlay
Saliency
ERECT
R
0.200
0.175
0.150
0.125
0.100
0.075
0.050
0.025
0.000

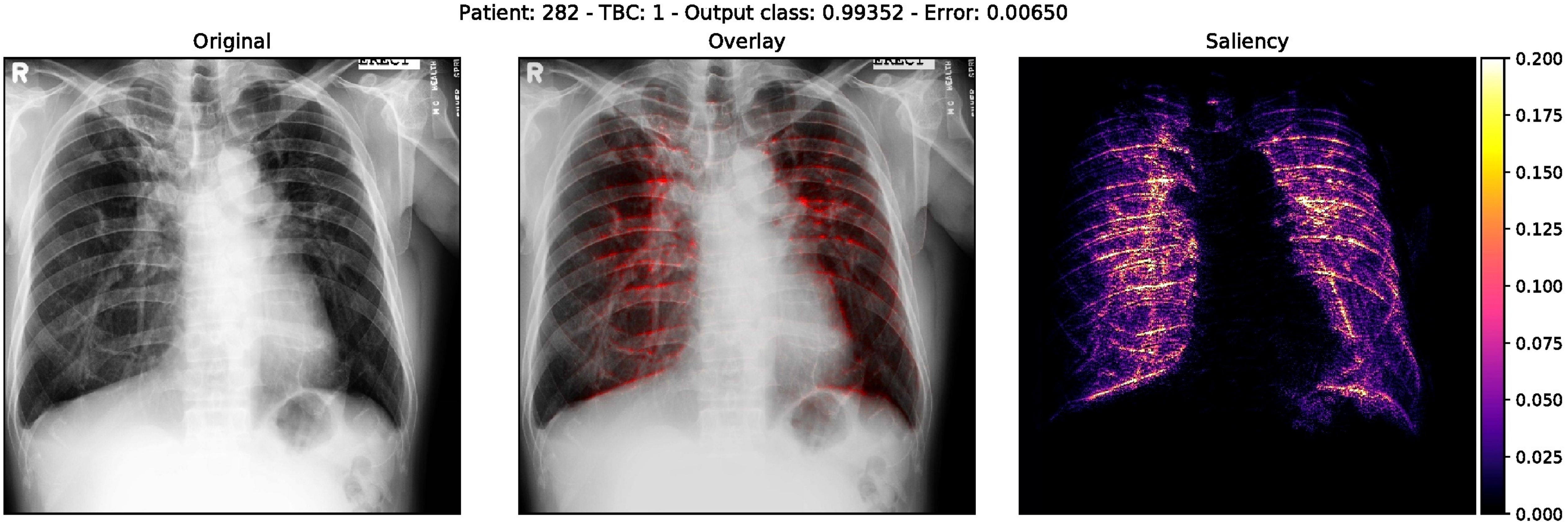
Patient: 282 - TBC: 1 - Output class: 0.99352 - Error: 0.00650
Original
Overlay
Saliency
R
0.200
0.175
0.150
0.125
0.100
0.075
0.050
0.025
0.000

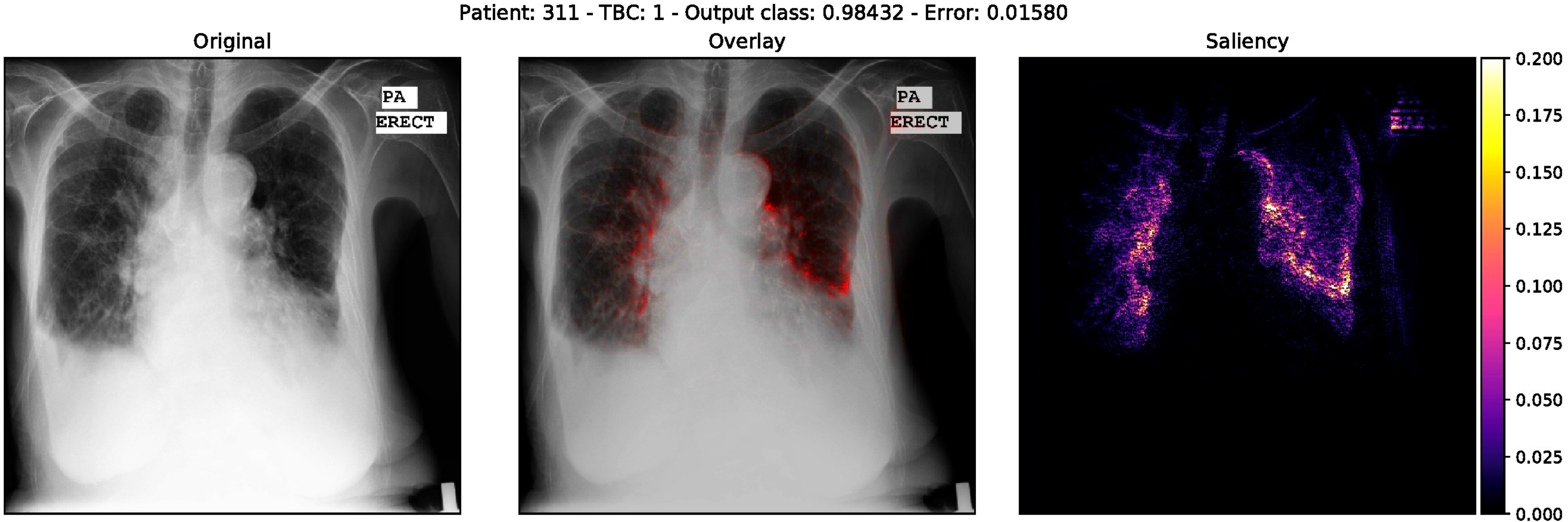
Patient: 311 - TBC: 1 - Output class: 0.98432 - Error: 0.01580
Original
Overlay
Saliency
PA
ERECT
PA
ERECT
0.200
0.175
0.150
0.125
0.100
0.075
0.050
0.025
0.000

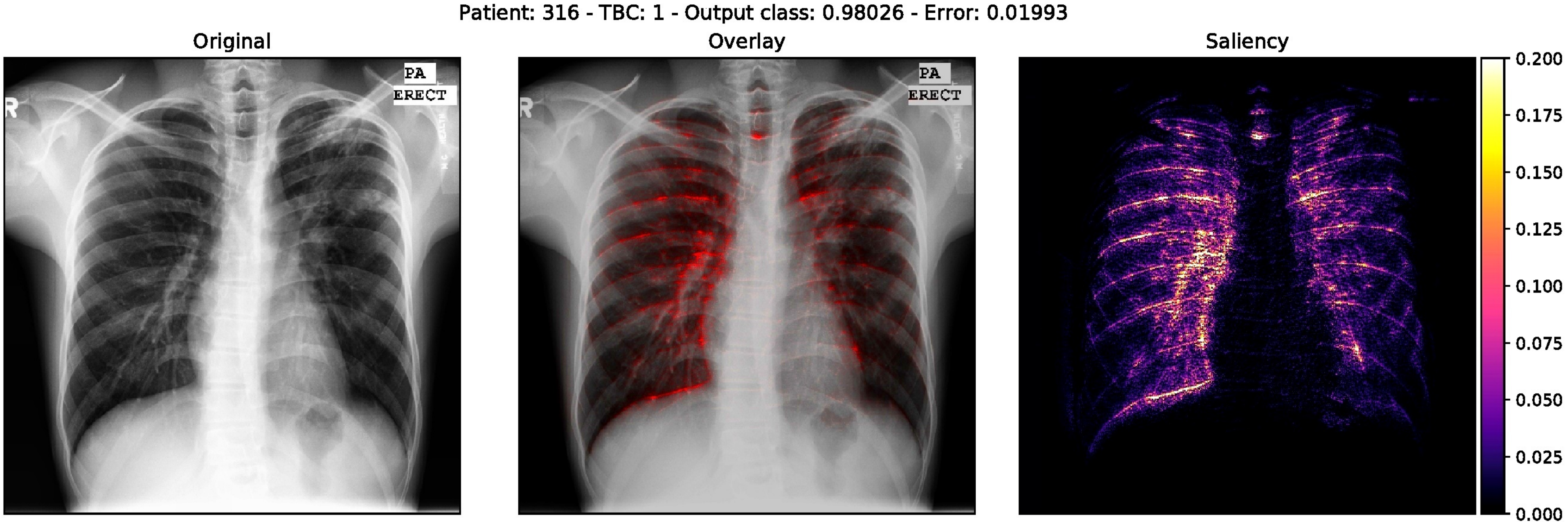

Patient: 316 - TBC: 1 - Output class: 0.98026 - Error: 0.01993
Original
Overlay
Saliency
PA
ERECT
R
0.200
0.175
0.150
0.125
0.100
0.075
0.050
0.025
0.000

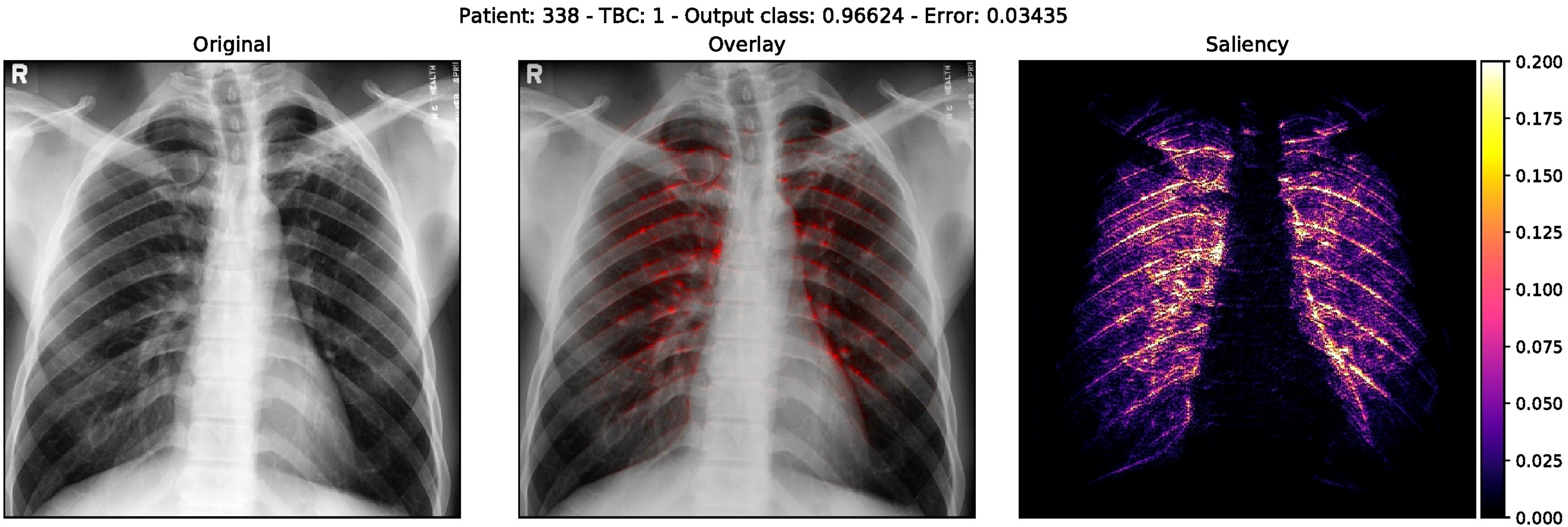
Patient: 338 - TBC: 1 - Output class: 0.96624 - Error: 0.03435
Original
Overlay
Saliency
R
R
0.200
0.175
0.150
0.125
0.100
0.075
0.050
0.025
0.000

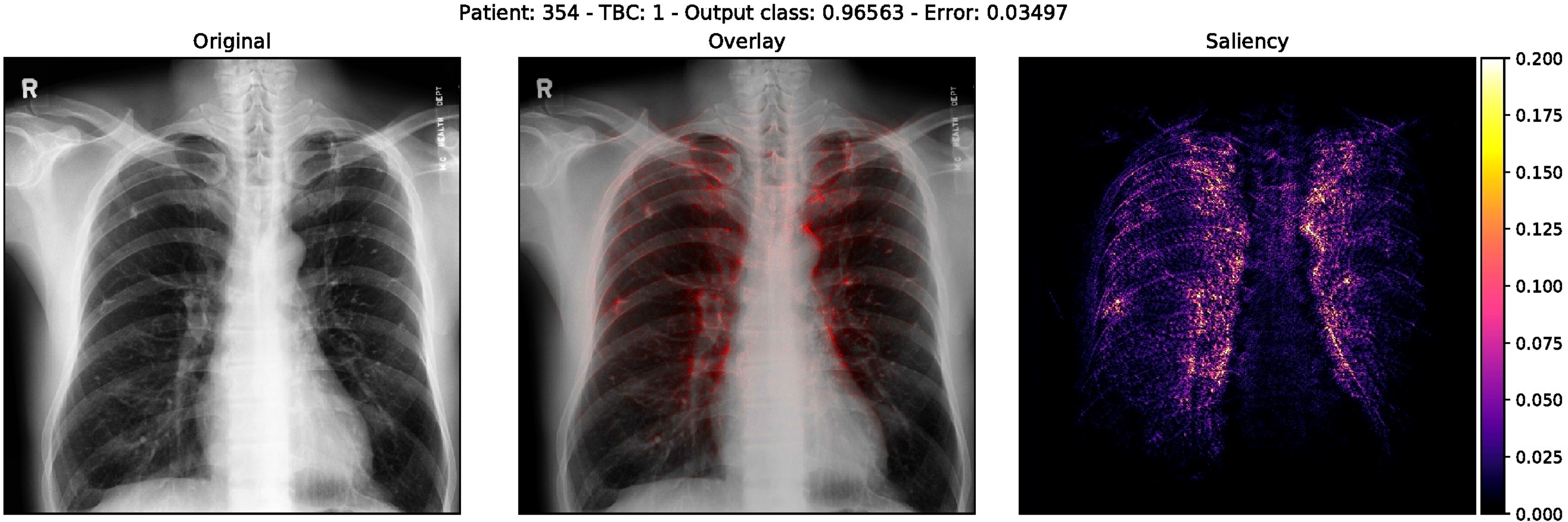
Patient: 354 - TBC: 1 - Output class: 0.96563 - Error: 0.03497
Original
Overlay
Saliency
R
0.200
0.175
0.150
0.125
0.100
0.075
0.050
0.025
0.000

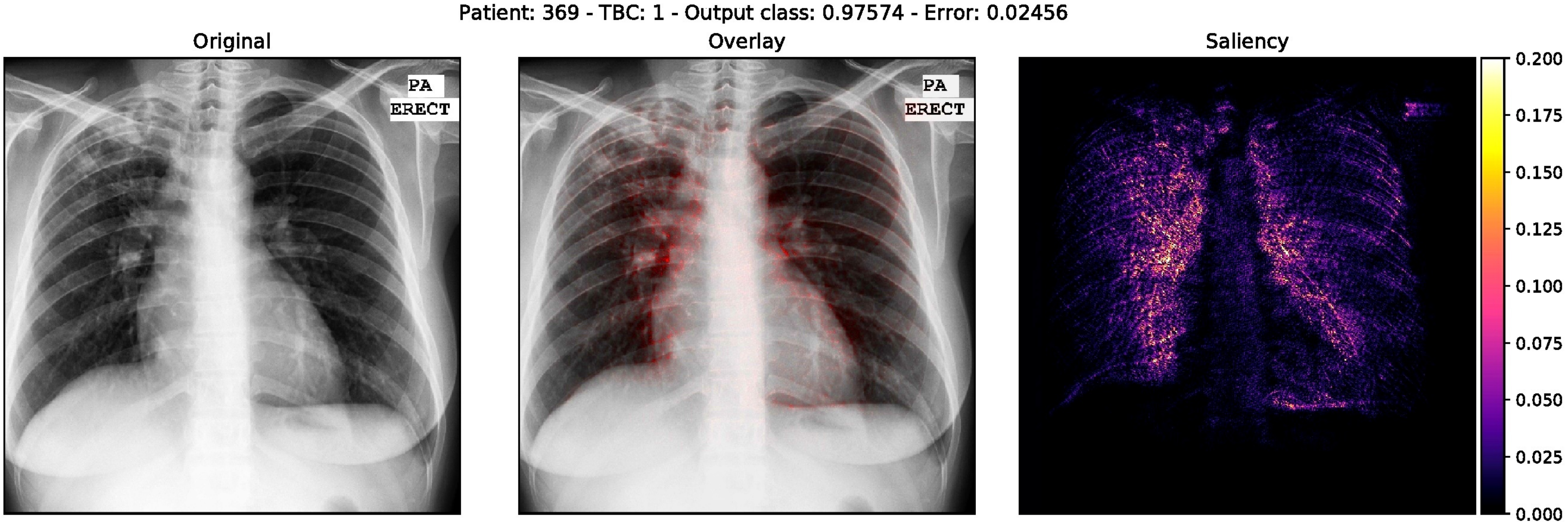

Patient: 369 - TBC: 1 - Output class: 0.97574 - Error: 0.02456
Original
Overlay
Saliency
PA
ERECT
PA
ERECT
0.200
0.175
0.150
0.125
0.100
0.075
0.050
0.025
0.000

True negatives

Patient: 1 - TBC: 0 - Output class: 0.00240 - Error: 0.00240

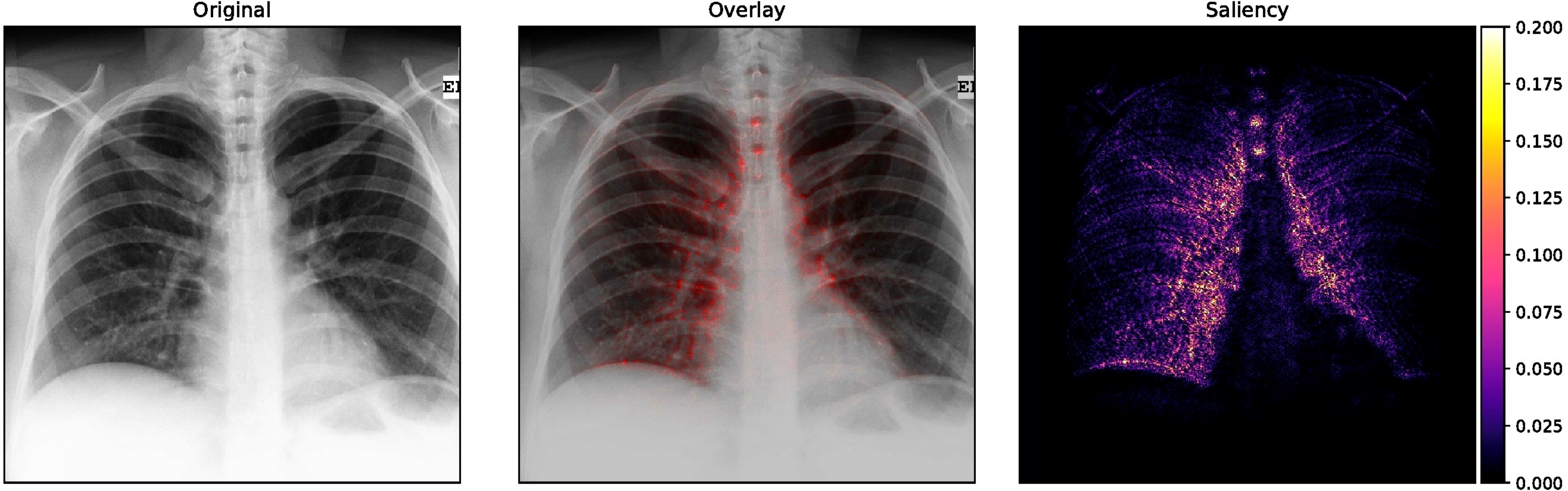

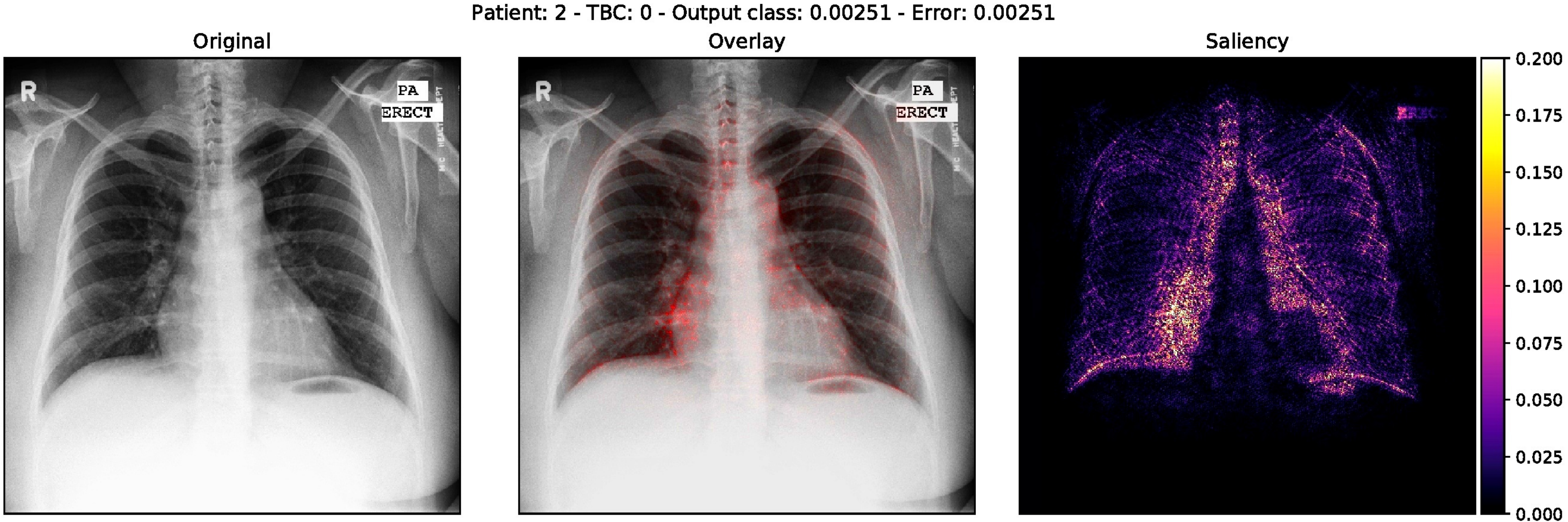
Patient: 2 - TBC: 0 - Output class: 0.00251 - Error: 0.00251
Original
Overlay
Saliency
R
PA
ERECT
0.200
0.175
0.150
0.125
0.100
0.075
0.050
0.025
0.000

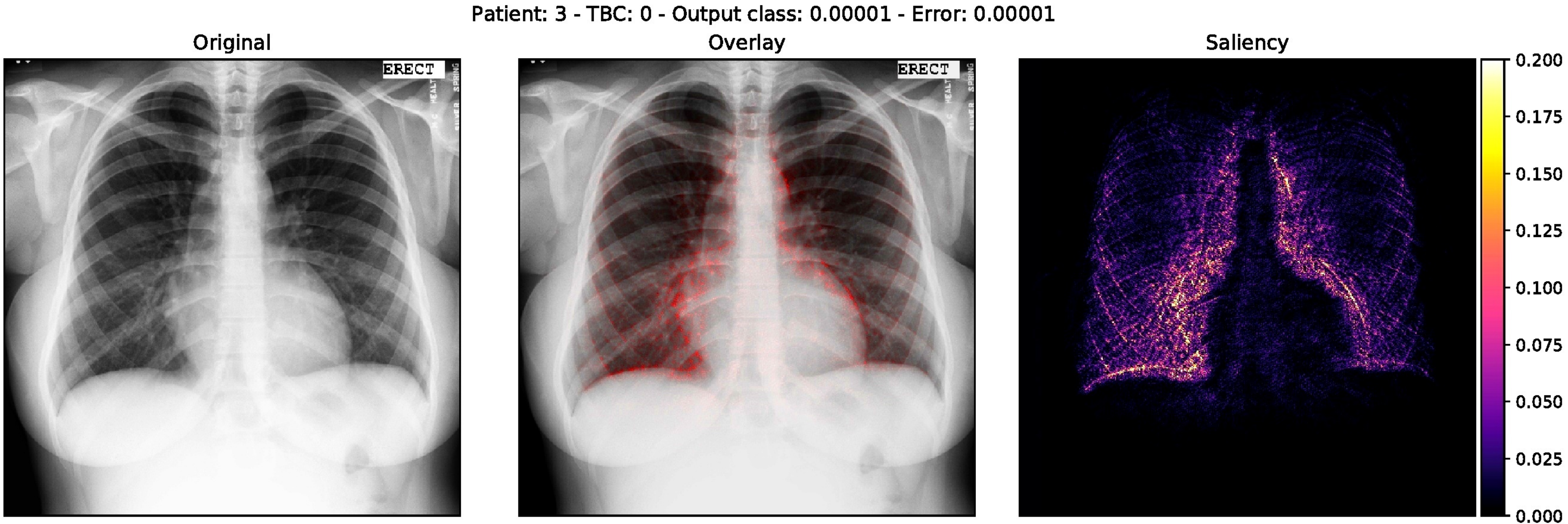
Patient: 3 - TBC: 0 - Output class: 0.00001 - Error: 0.00001
Original
Overlay
Saliency
ERECT
ERECT
0.200
0.175
0.150
0.125
0.100
0.075
0.050
0.025
0.000

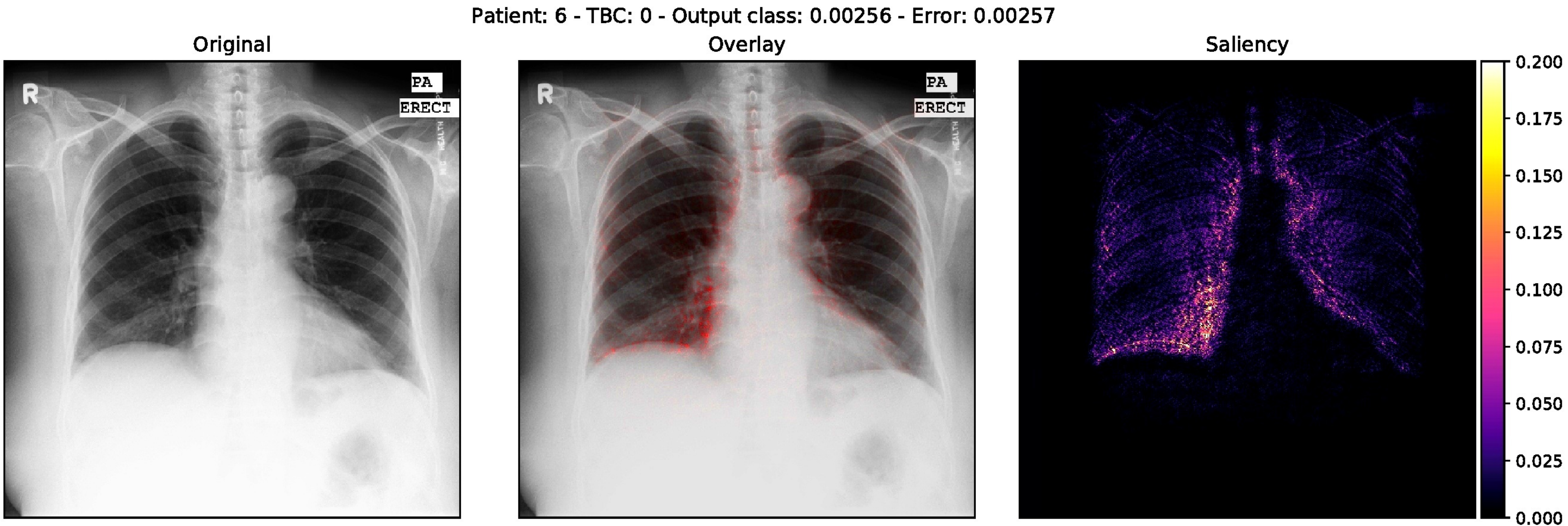

Patient: 6 - TBC: 0 - Output class: 0.00256 - Error: 0.00257
Original
Overlay
Saliency
R
PA
ERECT
0.200
0.175
0.150
0.125
0.100
0.075
0.050
0.025
0.000

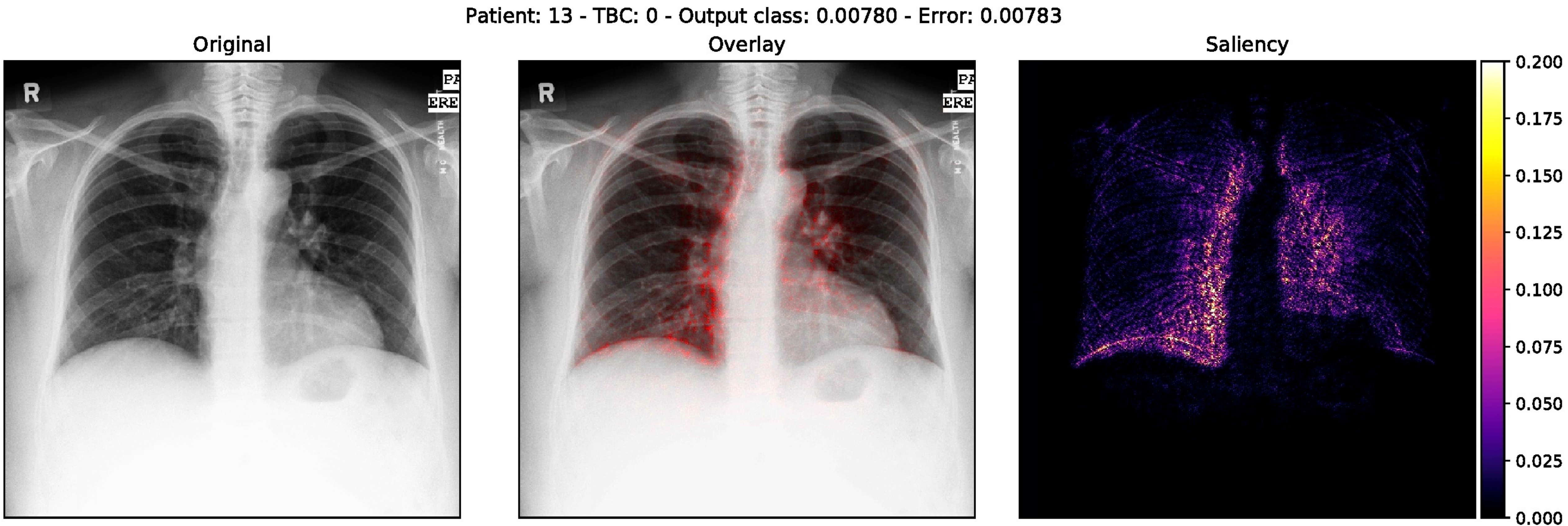
Patient: 13 - TBC: 0 - Output class: 0.00780 - Error: 0.00783
Original
Overlay
Saliency
0.200
0.175
0.150
0.125
0.100
0.075
0.050
0.025
0.000

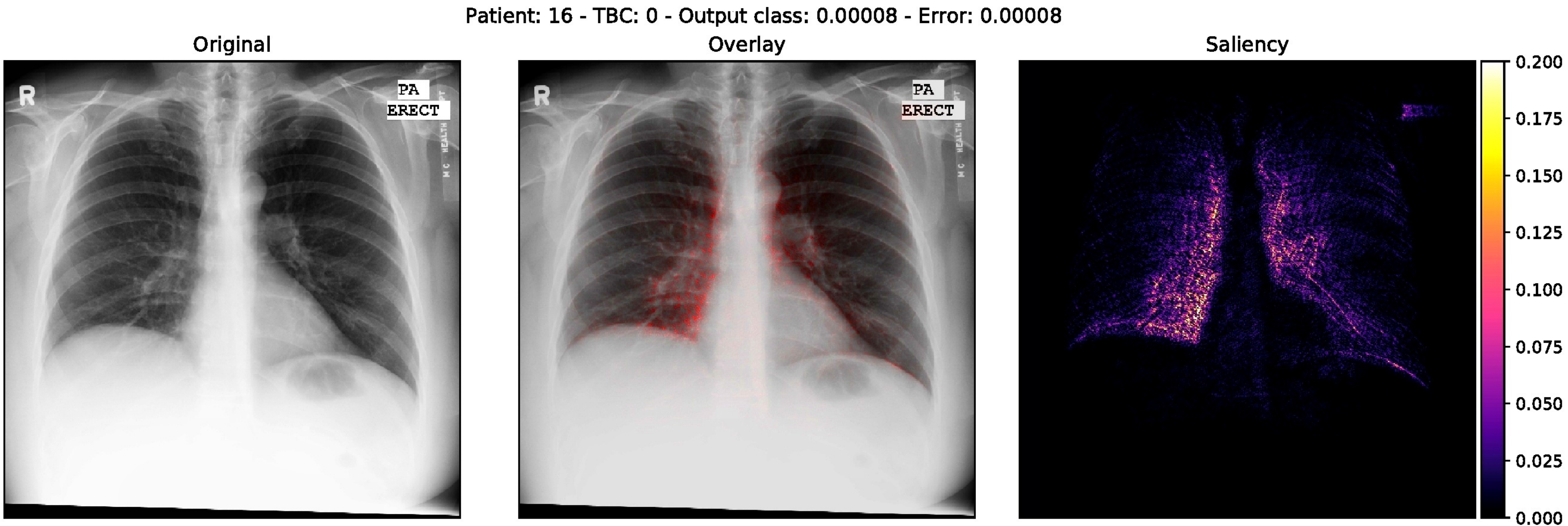
Patient: 16 - TBC: 0 - Output class: 0.00008 - Error: 0.00008
Original
Overlay
Saliency
R
PA
ERECT
0.200
0.175
0.150
0.125
0.100
0.075
0.050
0.025
0.000

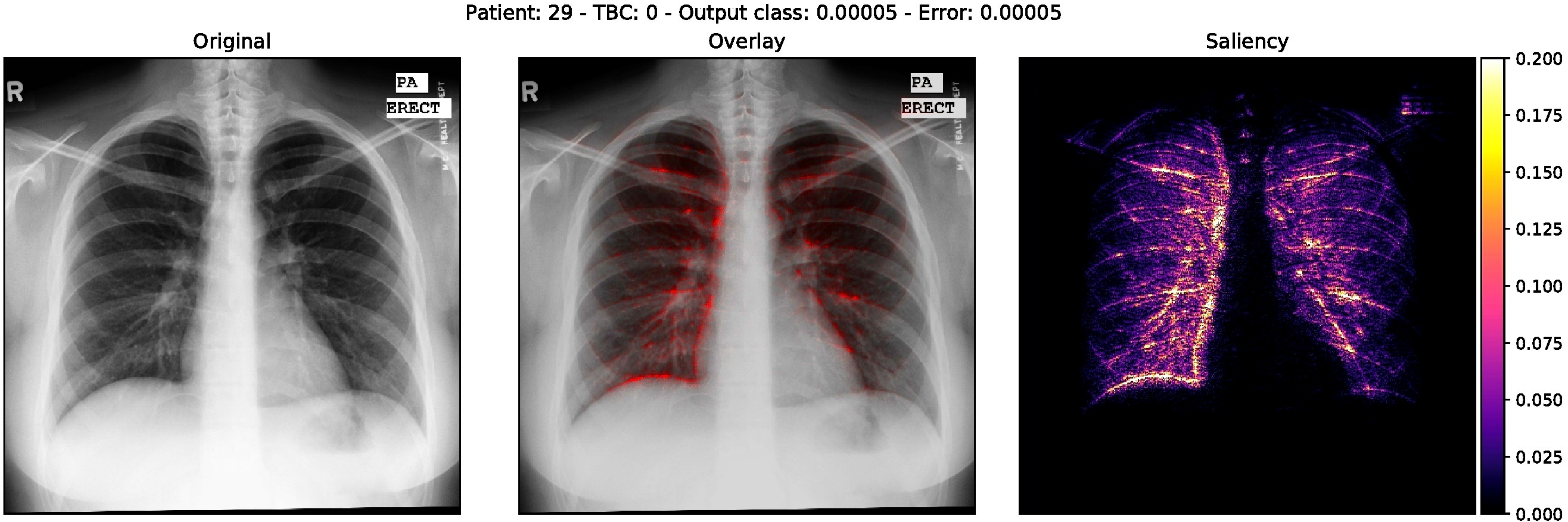
Patient: 29 - TBC: 0 - Output class: 0.00005 - Error: 0.00005
Original
Overlay
Saliency
R
PA
ERECT
0.200
0.175
0.150
0.125
0.100
0.075
0.050
0.025
0.000

Patient: 41 - TBC: 0 - Output class: 0.00078 - Error: 0.00078

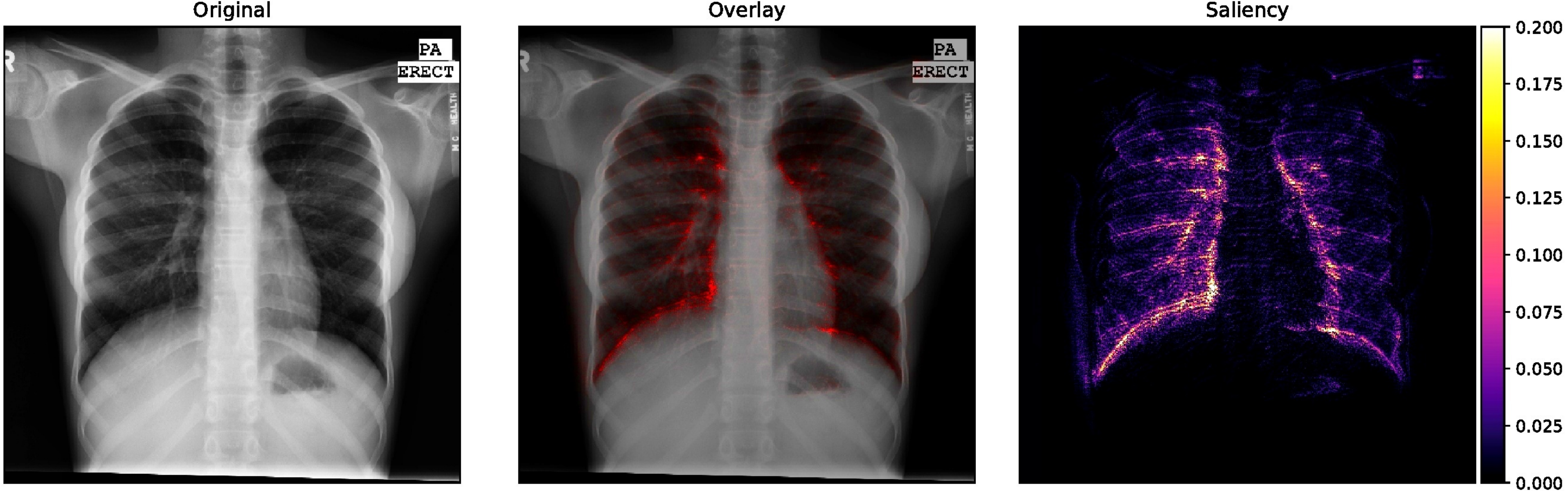

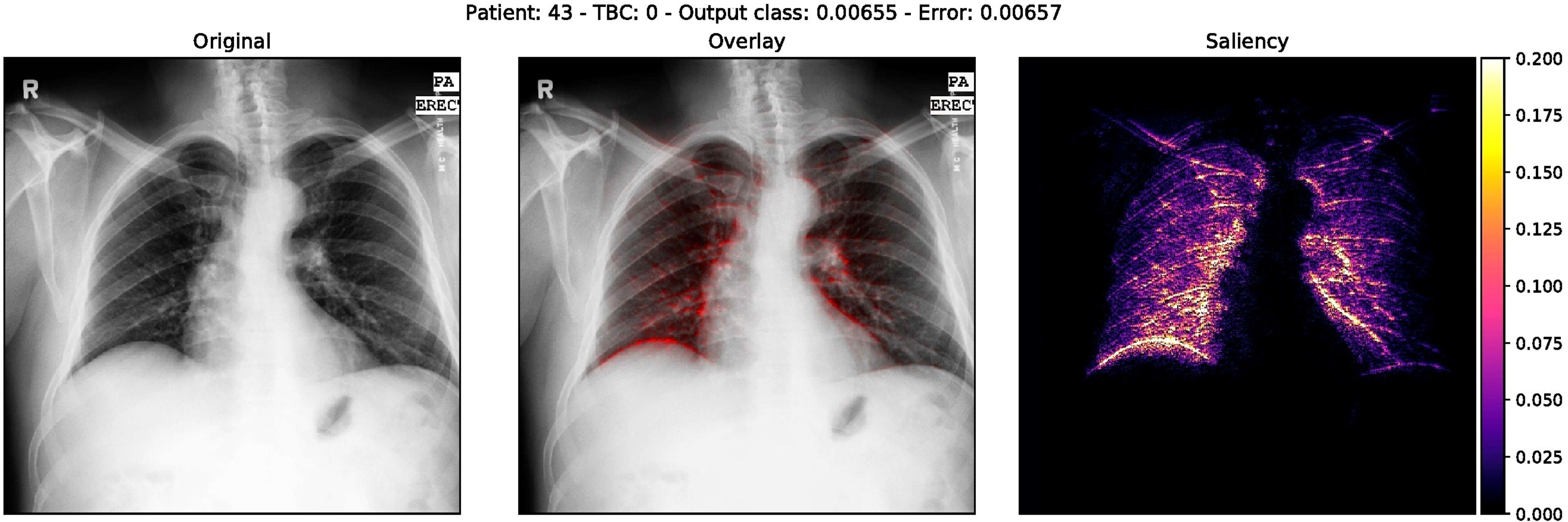

Patient: 43 - TBC: 0 - Output class: 0.00655 - Error: 0.00657
Original
Overlay
Saliency
R
PA
EREC
0.200
0.175
0.150
0.125
0.100
0.075
0.050
0.025
0.000

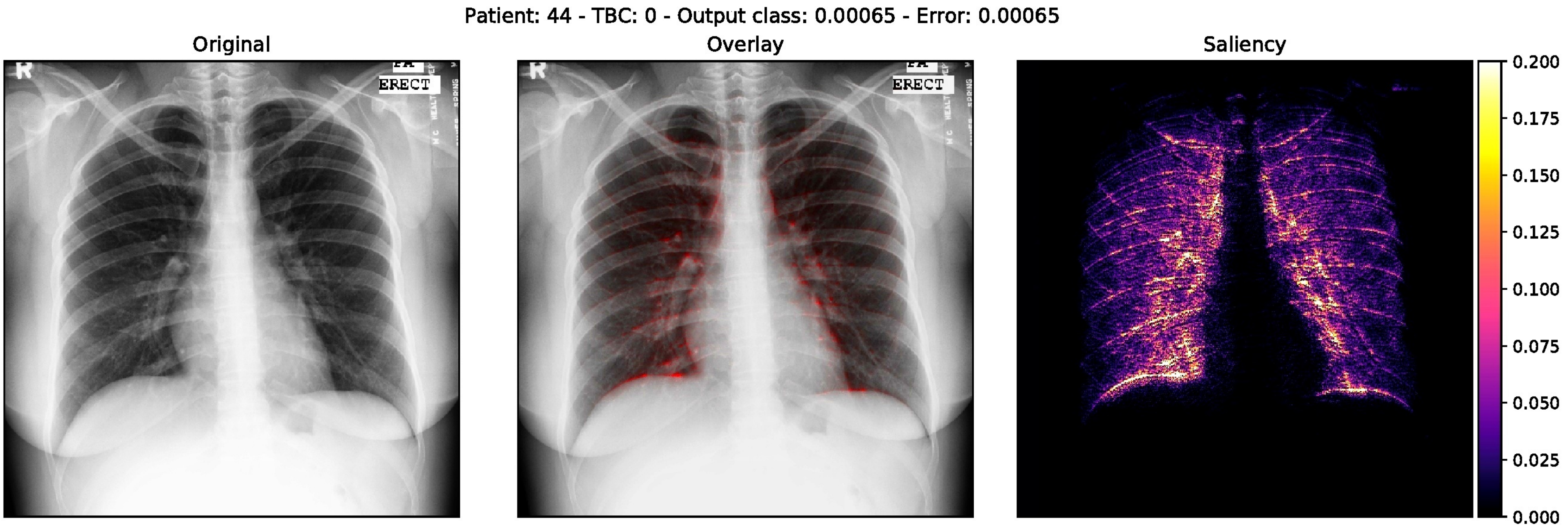
Patient: 44 - TBC: 0 - Output class: 0.00065 - Error: 0.00065
Original
Overlay
Saliency
R
ERECT
0.200
0.175
0.150
0.125
0.100
0.075
0.050
0.025
0.000

False positives

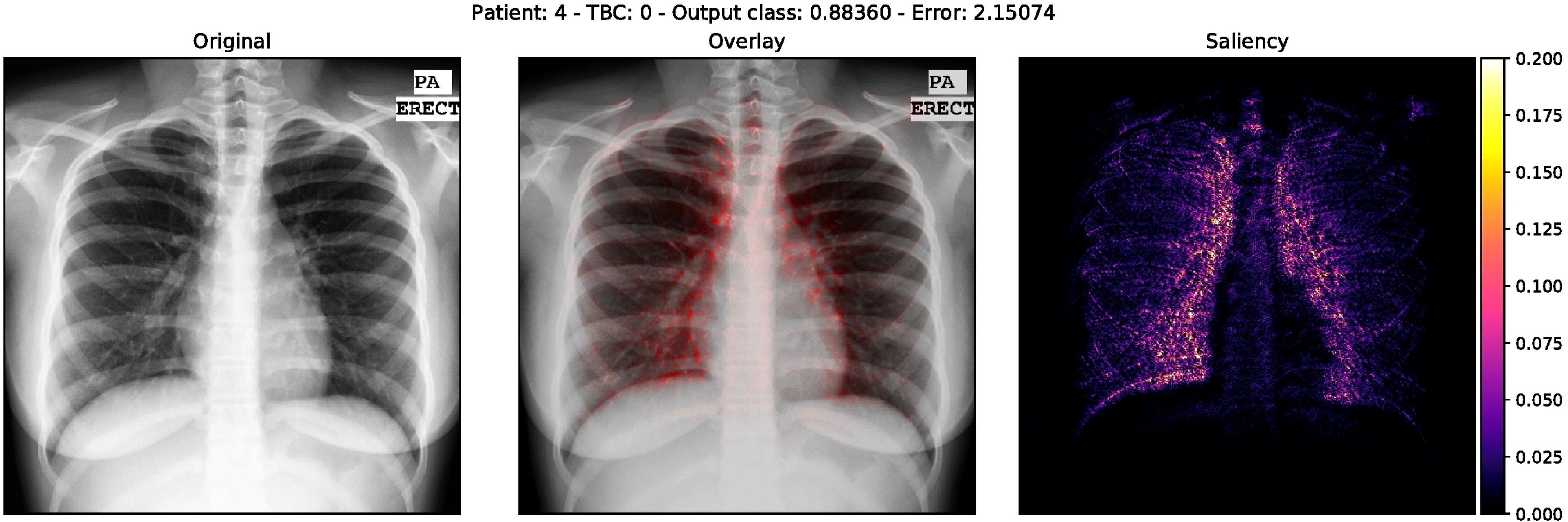

Patient: 4 - TBC: 0 - Output class: 0.88360 - Error: 2.15074
Original
Overlay
Saliency
PA
ERECT
0.200
0.175
0.150
0.125
0.100
0.075
0.050
0.025
0.000

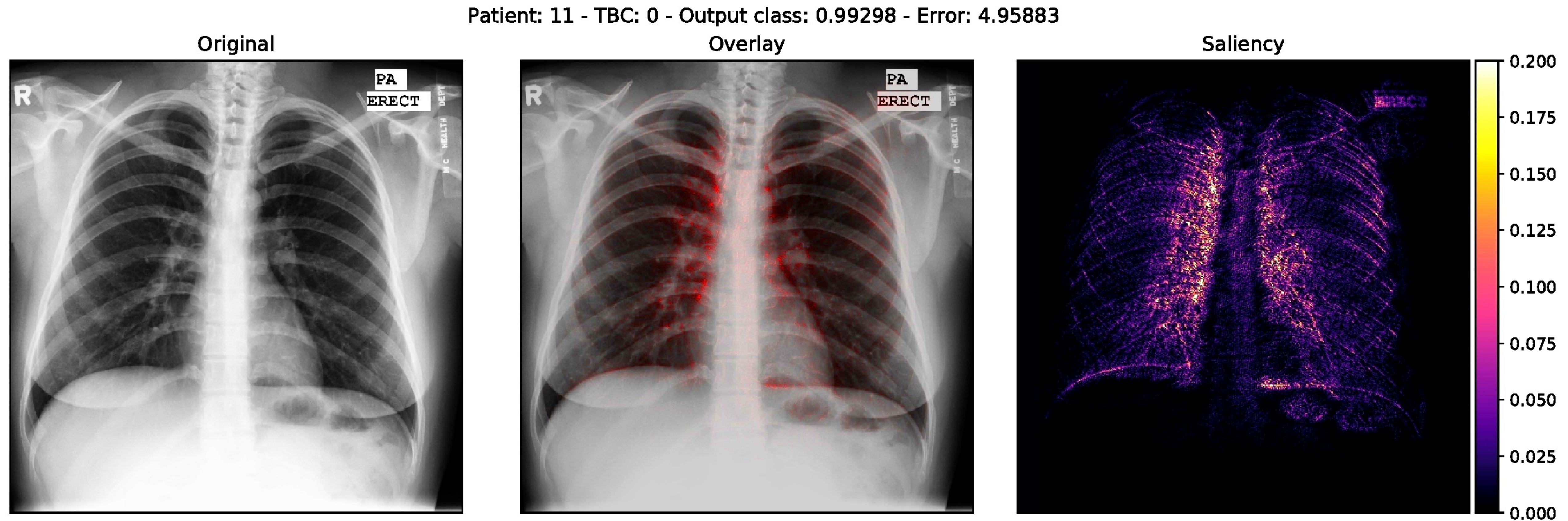
Patient: 11 - TBC: 0 - Output class: 0.99298 - Error: 4.95883
Original
Overlay
Saliency
PA
ERECT
R
0.200
0.175
0.150
0.125
0.100
0.075
0.050
0.025
0.000

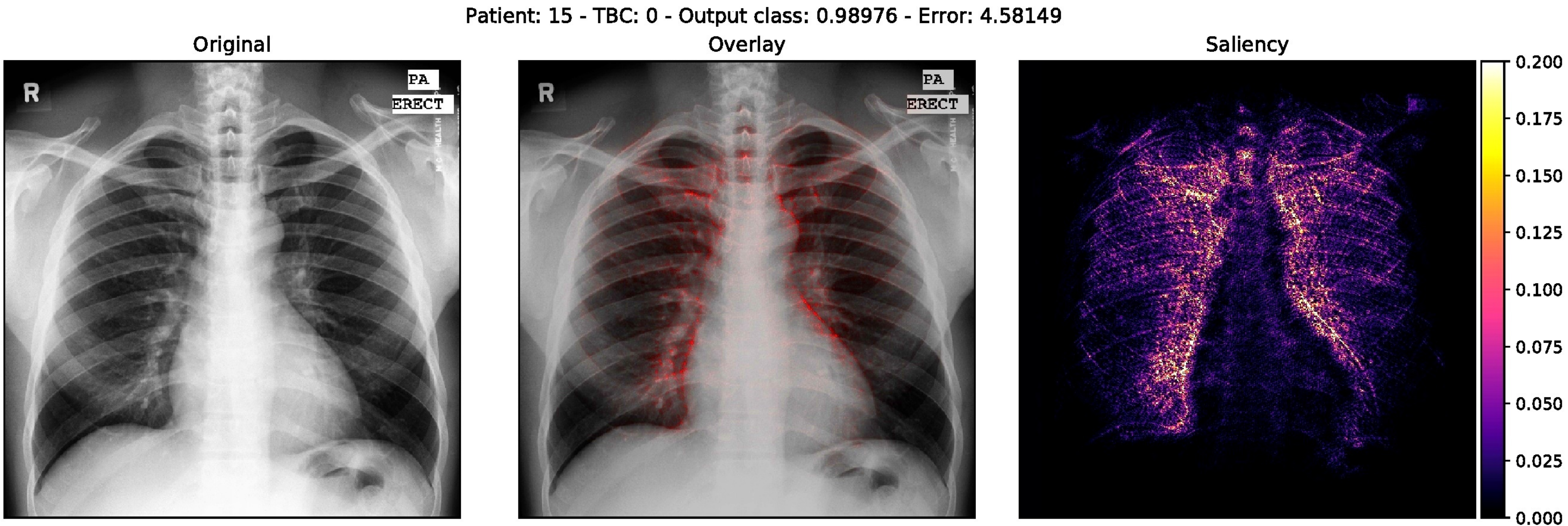
Patient: 15 - TBC: 0 - Output class: 0.98976 - Error: 4.58149
Original
Overlay
Saliency
R
PA
ERECT
0.200
0.175
0.150
0.125
0.100
0.075
0.050
0.025
0.000

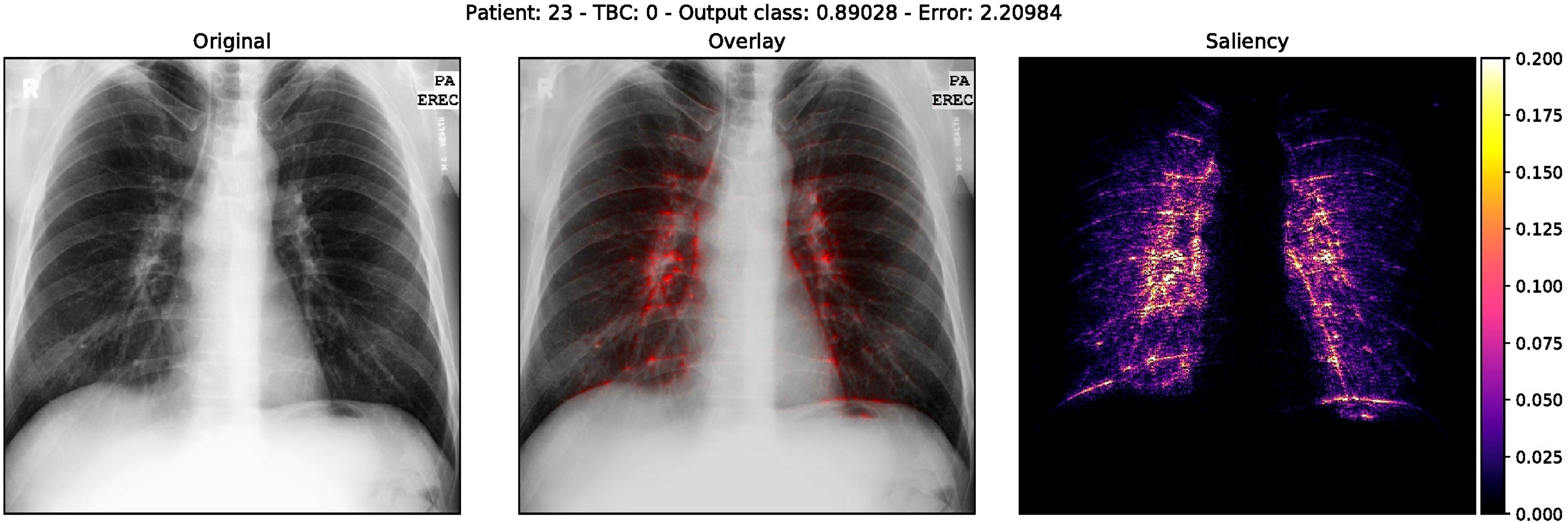
Patient: 23 - TBC: 0 - Output class: 0.89028 - Error: 2.20984
Original
Overlay
Saliency
R
PA
EREC
0.200
0.175
0.150
0.125
0.100
0.075
0.050
0.025
0.000

Patient: 30 - TBC: 0 - Output class: 0.91033 - Error: 2.41163

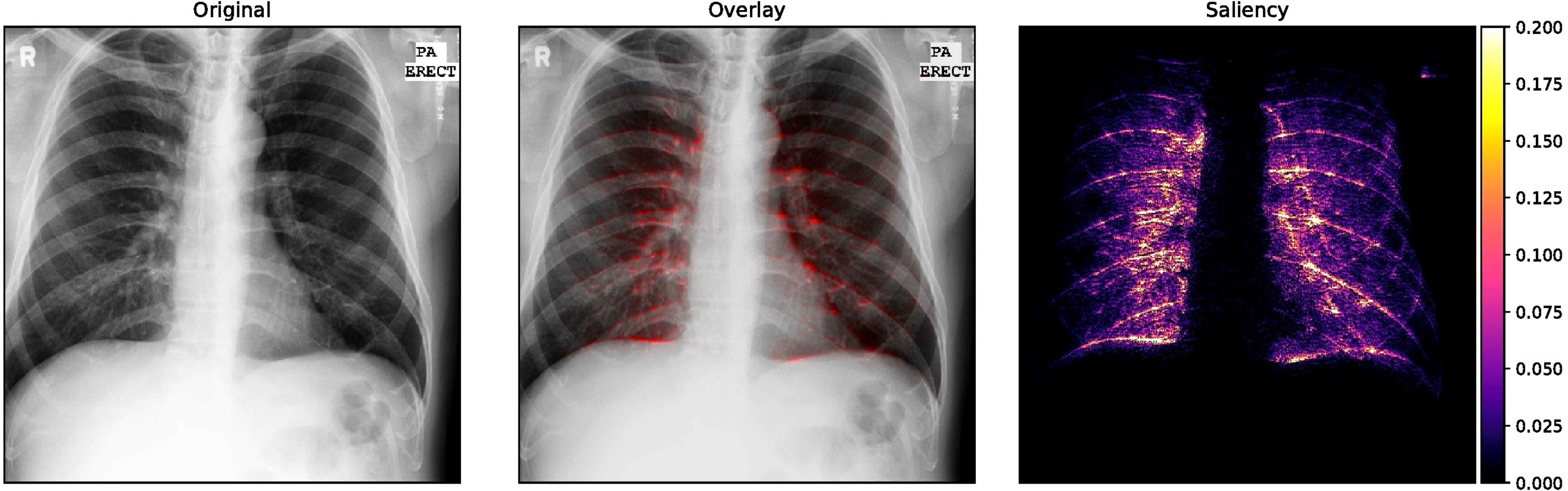

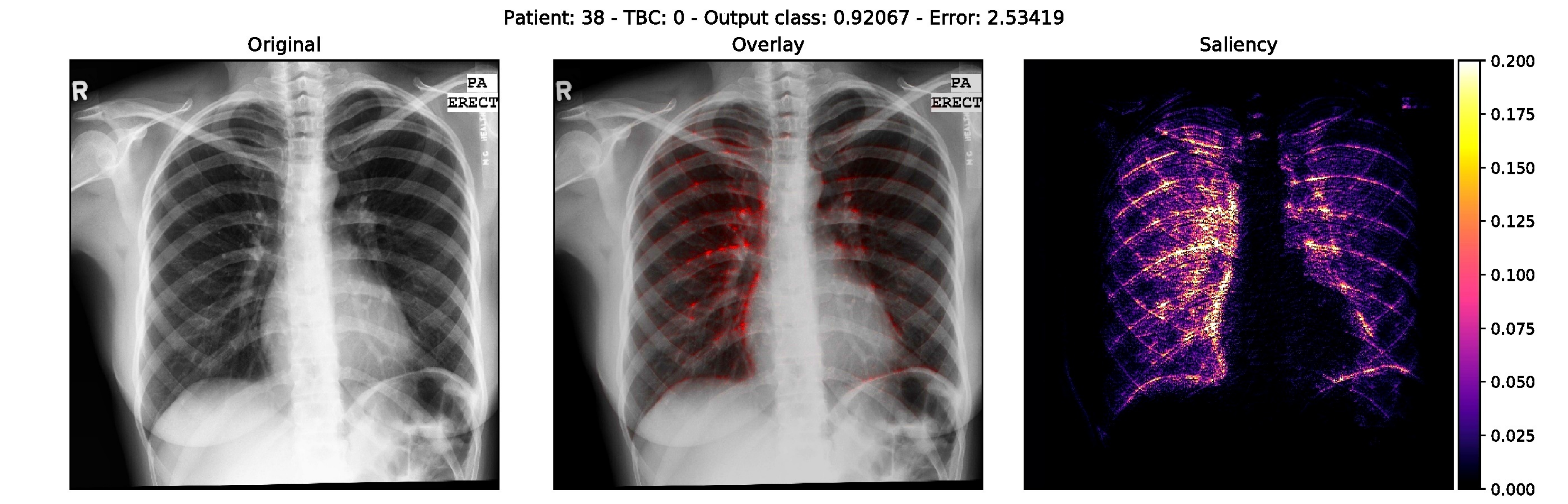
Patient: 38 - TBC: 0 - Output class: 0.92067 - Error: 2.53419
Original
Overlay
Saliency
R
PA
ERECT
0.200
0.175
0.150
0.125
0.100
0.075
0.050
0.025
0.000

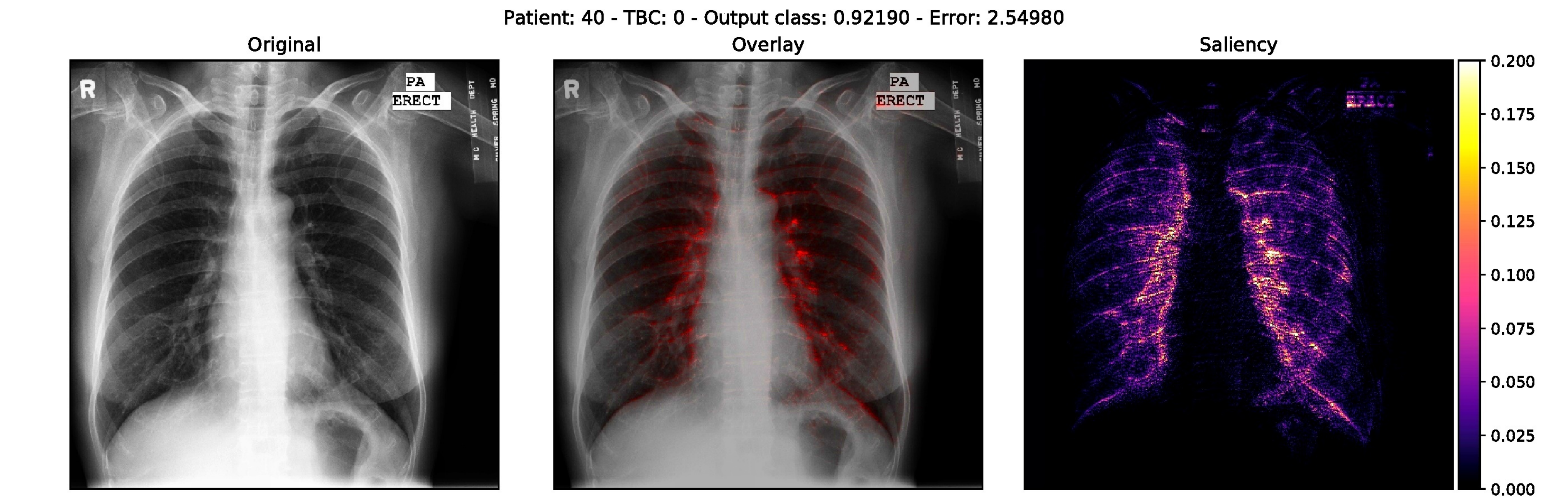
Patient: 40 - TBC: 0 - Output class: 0.92190 - Error: 2.54980
Original
Overlay
Saliency
R
PA
ERECT
0.200
0.175
0.150
0.125
0.100
0.075
0.050
0.025
0.000

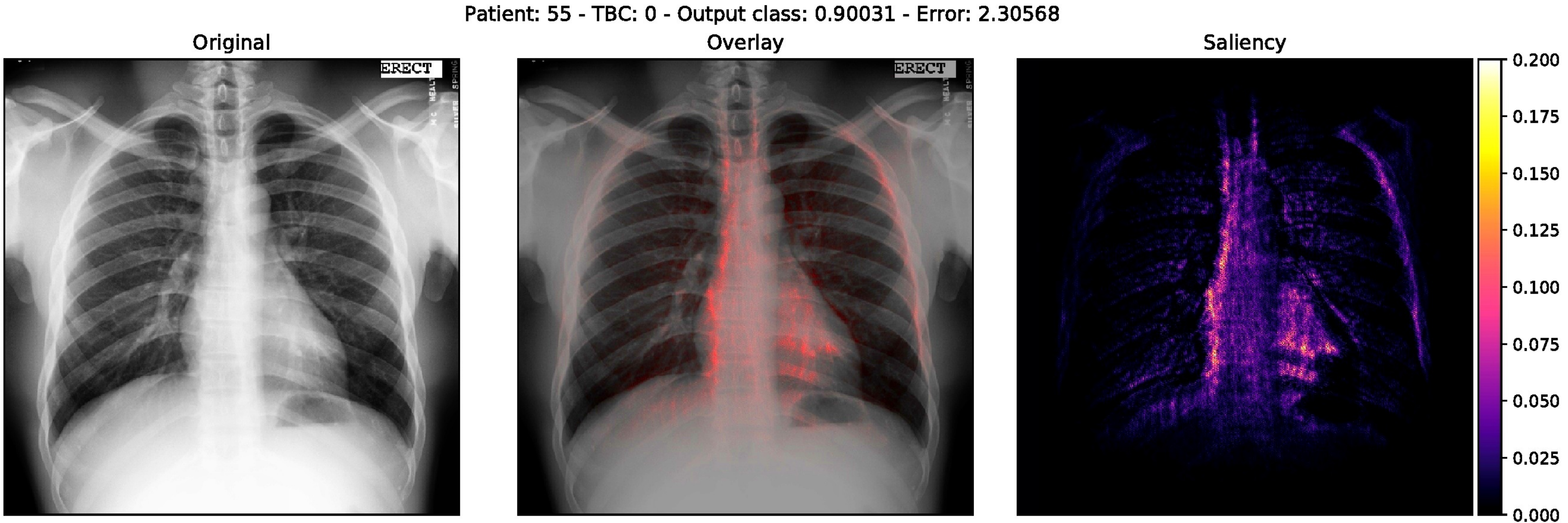
Patient: 55 - TBC: 0 - Output class: 0.90031 - Error: 2.30568
Original
Overlay
Saliency
ERECT
ERECT
0.200
0.175
0.150
0.125
0.100
0.075
0.050
0.025
0.000

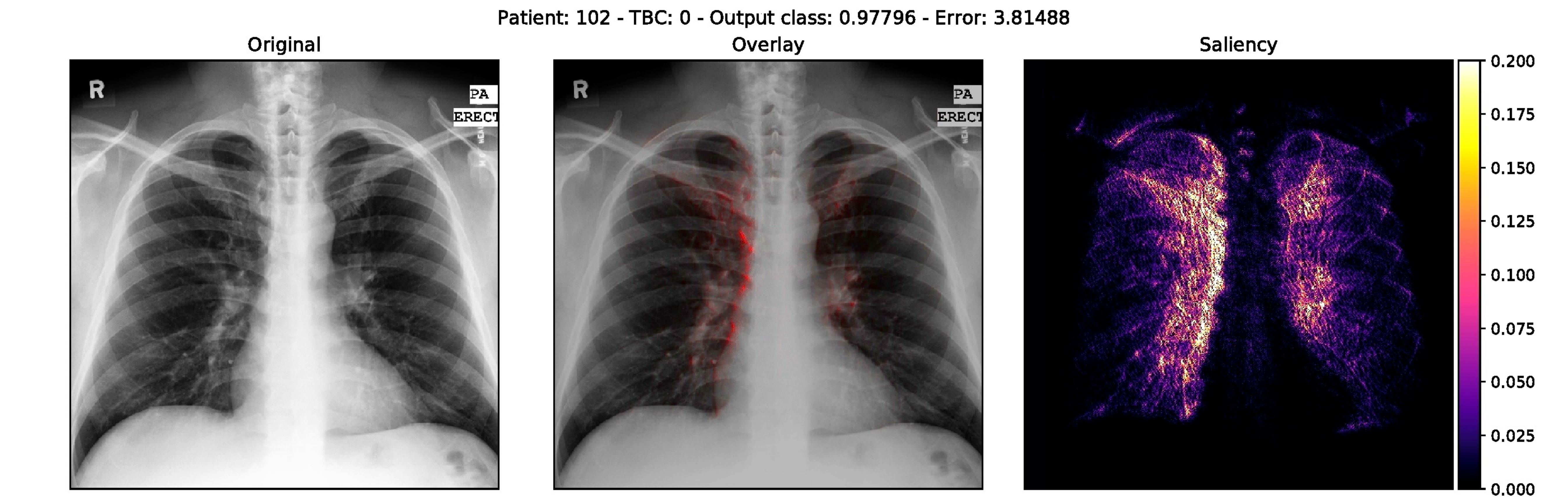

Patient: 102 - TBC: 0 - Output class: 0.97796 - Error: 3.81488
Original
Overlay
Saliency
R
PA
ERECT
0.200
0.175
0.150
0.125
0.100
0.075
0.050
0.025
0.000

False negatives

Patient: 104 - TBC: 1 - Output class: 0.01206 - Error: 4.41749

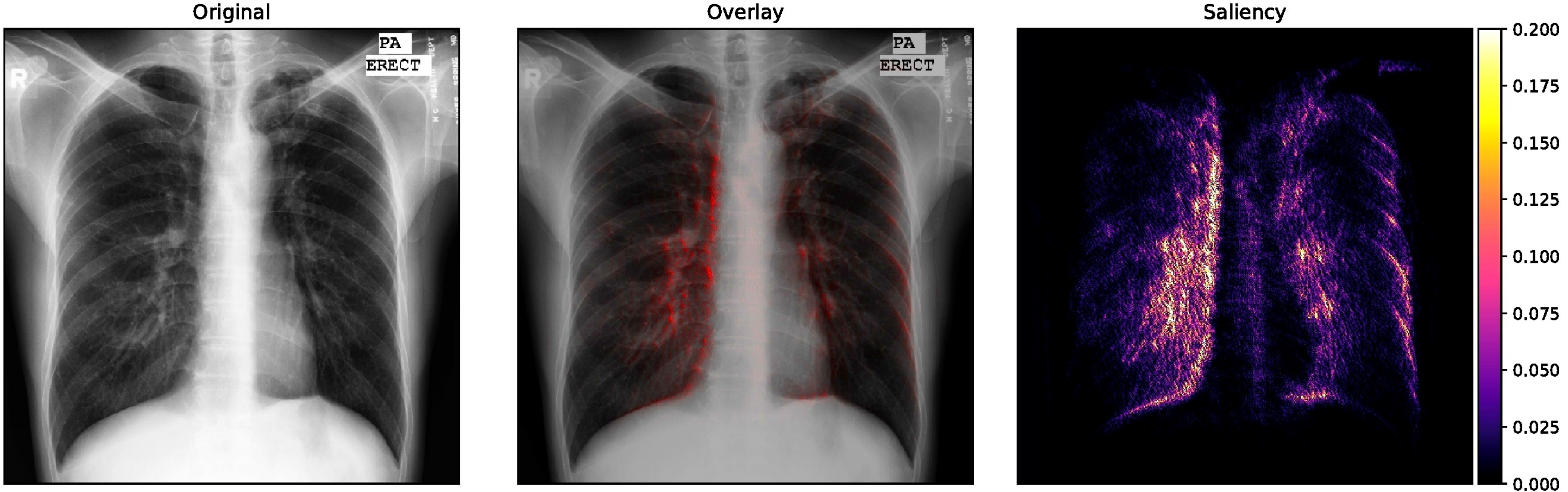

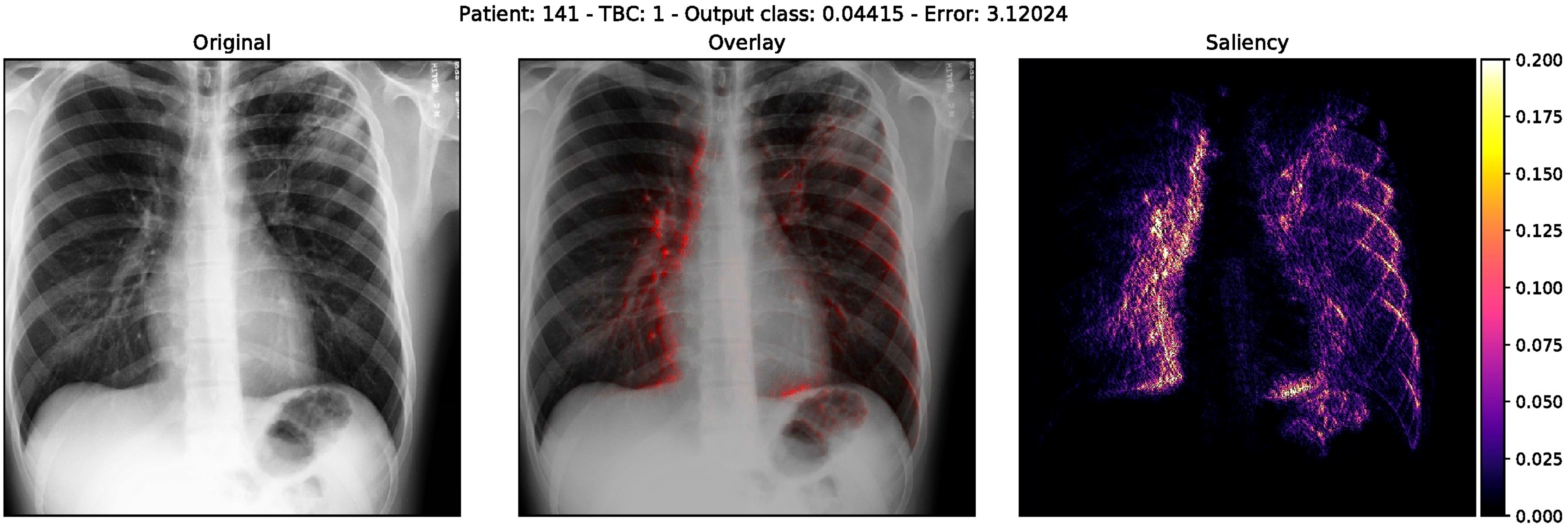
Patient: 141 - TBC: 1 - Output class: 0.04415 - Error: 3.12024
Original
Overlay
Saliency
0.200
0.175
0.150
0.125
0.100
0.075
0.050
0.025
0.000

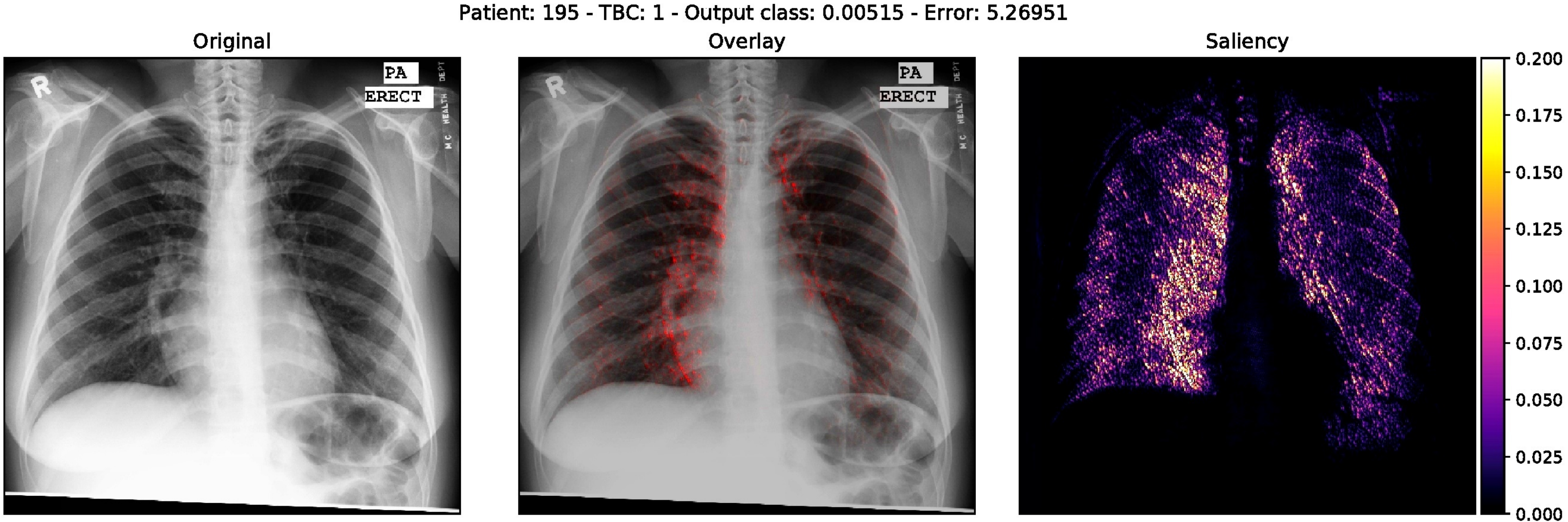

Patient: 195 - TBC: 1 - Output class: 0.00515 - Error: 5.26951
Original
Overlay
Saliency
R
PA
ERECT
0.200
0.175
0.150
0.125
0.100
0.075
0.050
0.025
0.000

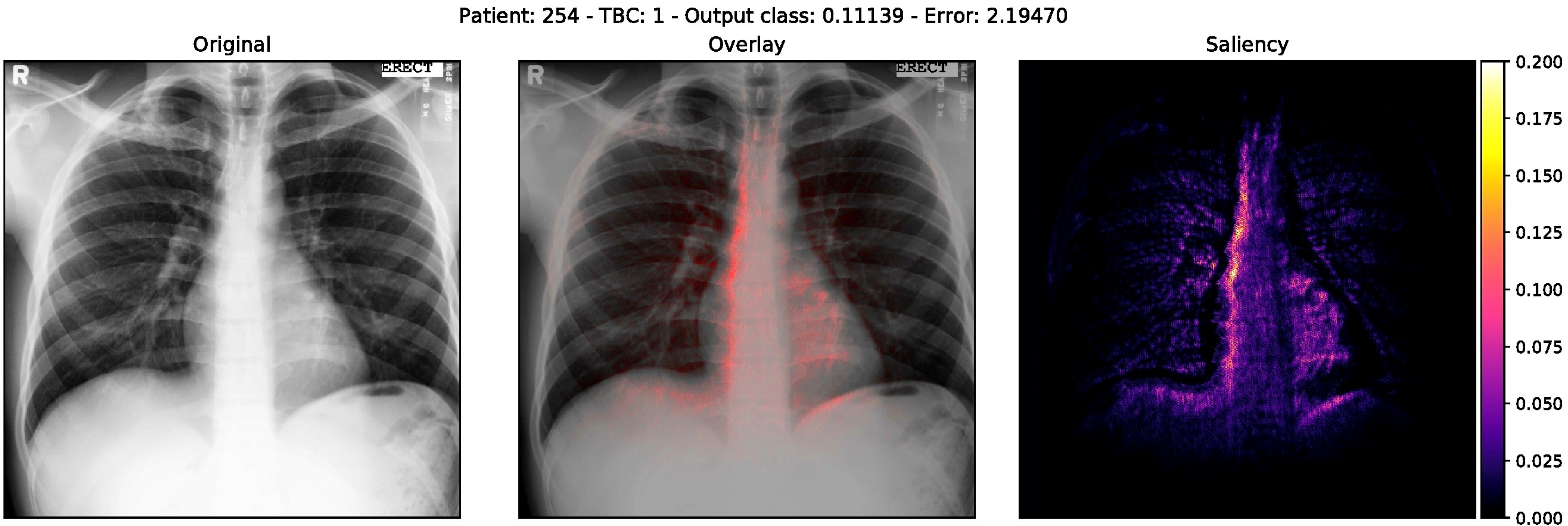
Patient: 254 - TBC: 1 - Output class: 0.11139 - Error: 2.19470
Original
Overlay
Saliency
R
ERECT
0.200
0.175
0.150
0.125
0.100
0.075
0.050
0.025
0.000

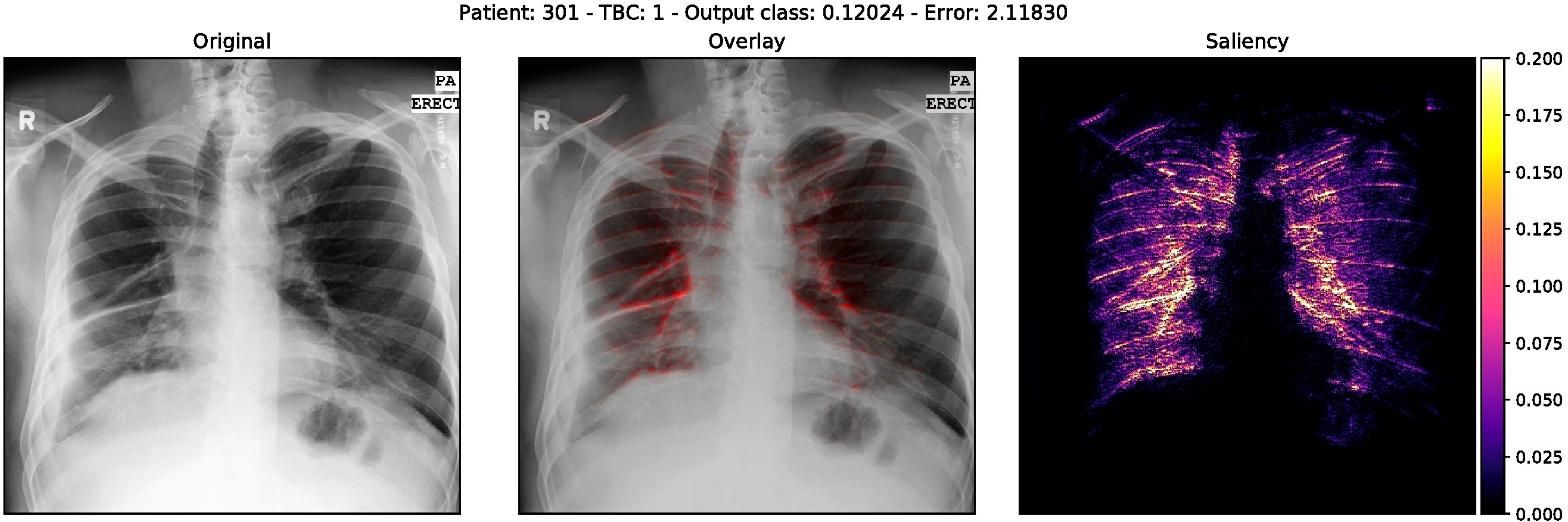
Patient: 301 - TBC: 1 - Output class: 0.12024 - Error: 2.11830
Original
Overlay
Saliency
PA
ERECT
R
0.200
0.175
0.150
0.125
0.100
0.075
0.050
0.025
0.000

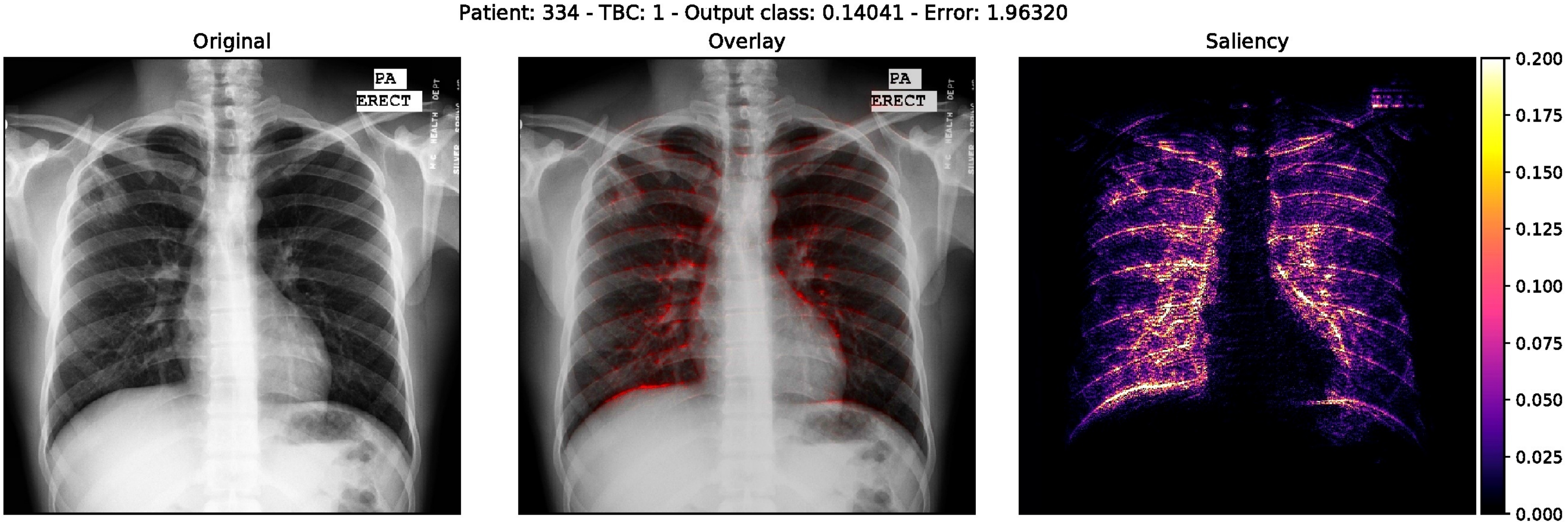

Patient: 334 - TBC: 1 - Output class: 0.14041 - Error: 1.96320
Original
Overlay
Saliency
PA
ERECT
0.200
0.175
0.150
0.125
0.100
0.075
0.050
0.025
0.000

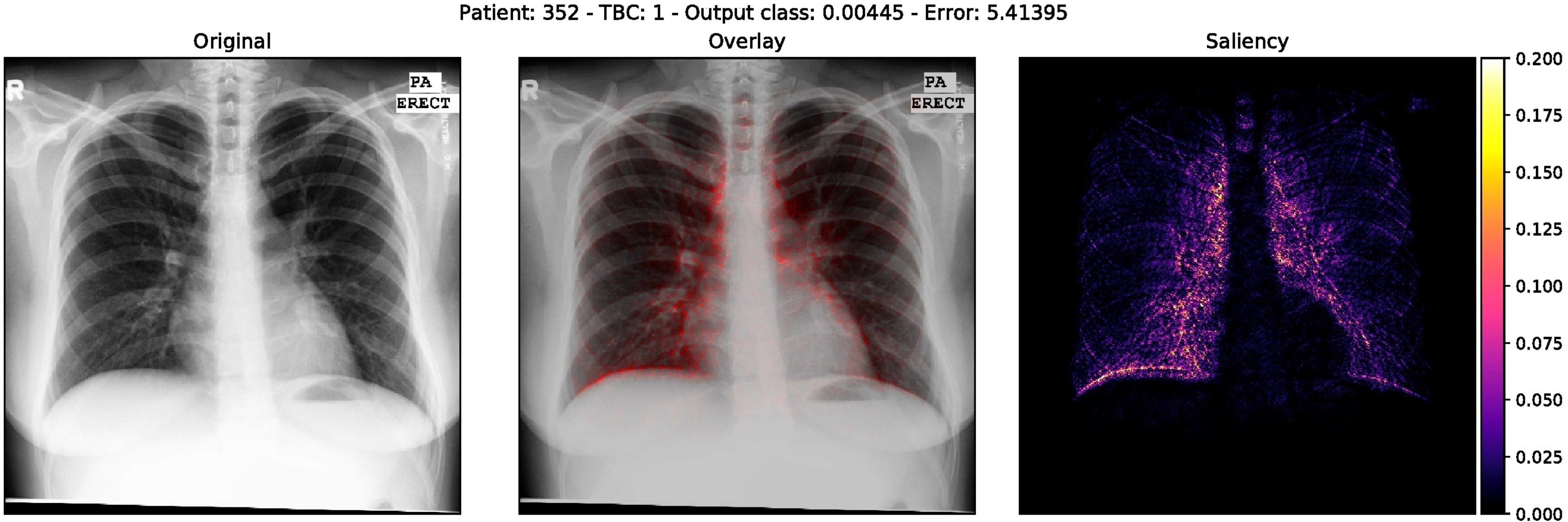
Patient: 352 - TBC: 1 - Output class: 0.00445 - Error: 5.41395
Original
Overlay
Saliency
R
PA
ERECT
0.200
0.175
0.150
0.125
0.100
0.075
0.050
0.025
0.000

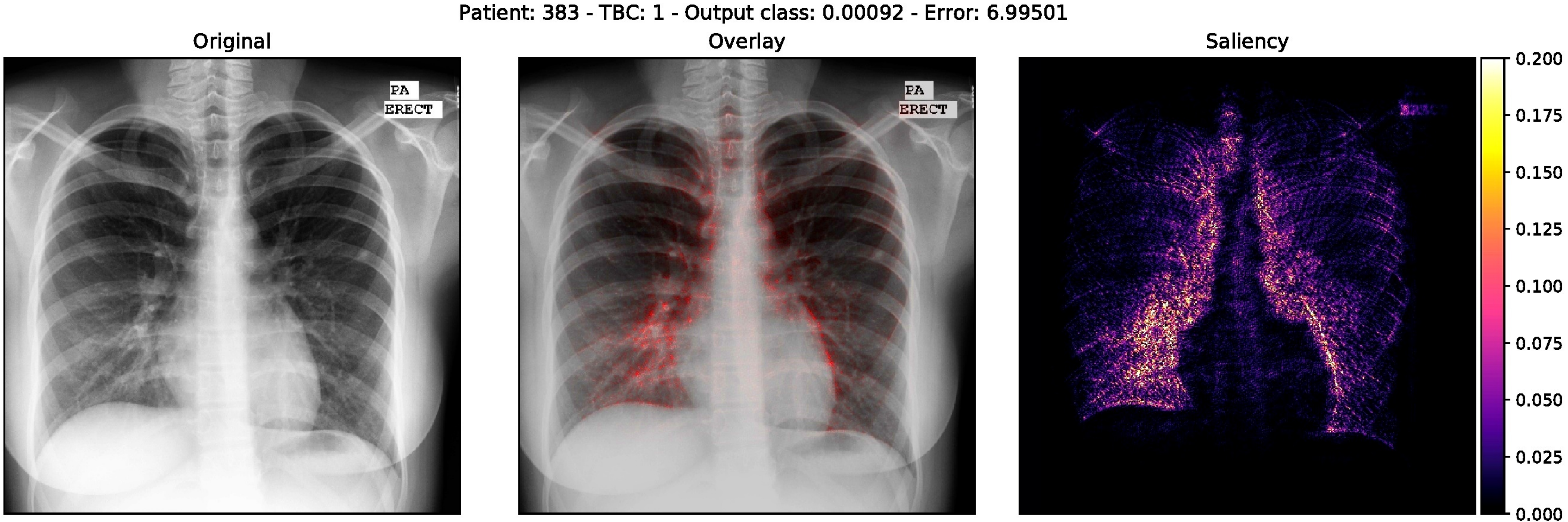
Patient: 383 - TBC: 1 - Output class: 0.00092 - Error: 6.99501
Original
Overlay
Saliency
PA
ERECT
PA
ERECT
0.200
0.175
0.150
0.125
0.100
0.075
0.050
0.025
0.000
